# Fluorescence-Based Activity Screening Assay Reveals Small Molecule Inhibitors of Vaccinia Virus mRNA Decapping Enzyme D9

**DOI:** 10.1021/acschembio.2c00049

**Published:** 2022-05-16

**Authors:** Marcelina Bednarczyk, Jessica K. Peters, Renata Kasprzyk, Jagoda Starek, Marcin Warminski, Tomasz Spiewla, Jeffrey S. Mugridge, John D. Gross, Jacek Jemielity, Joanna Kowalska

**Affiliations:** † Division of Biophysics, Institute of Experimental Physics, Faculty of Physics, University of Warsaw, Pasteura 5, Warsaw 02-093, Poland; ‡ Centre of New Technologies, University of Warsaw, Banacha 2C, Warsaw 02-097, Poland; § Department of Pharmaceutical Chemistry, University of California, San Francisco, California 94158, United States; ∥ Department of Chemistry & Biochemistry, University of Delaware, Newark, Delaware 19716, United States

## Abstract

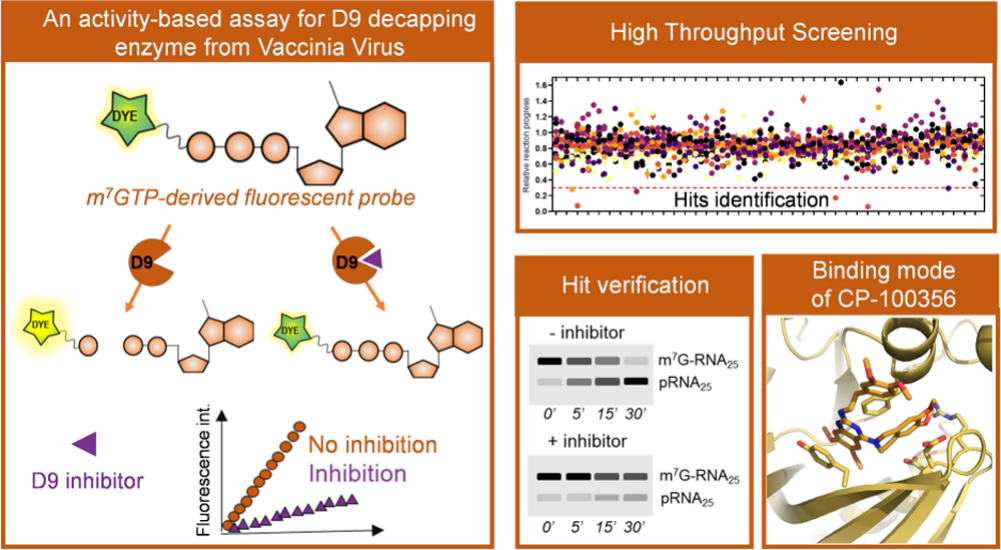

Vaccinia virus (VACV) represents a family of poxviruses, which possess their own decapping machinery as a part of their strategy to eliminate host mRNAs and evade the innate immune response. D9 is one of the two encoded VACV decapping enzymes that is responsible for cap removal from the 5′ end of both host mRNA transcripts and viral double-stranded RNAs. Little is known about the structural requirements for D9 inhibition by small molecules. Here, we identified a minimal D9 substrate and used it to develop a real-time fluorescence assay for inhibitor discovery and characterization. We screened a panel of nucleotide-derived substrate analogues and pharmacologically active candidates to identify several compounds with nano- and low micromolar IC_50_ values. m^7^GpppCH_2_p was the most potent nucleotide inhibitor (IC_50_ ∼ 0.08 μM), and seliciclib and CP-100356 were the most potent drug-like compounds (IC_50_ 0.57 and 2.7 μM, respectively). The hits identified through screening inhibited D9-catalyzed decapping of 26 nt RNA substrates but were not active toward VACV D10 or human decapping enzyme, Dcp1/2. The inhibition mode for one of the compounds (CP-100356) was elucidated based on the X-ray cocrystal structure, opening the possibility for structure-based design of novel D9 inhibitors and binding probes.

## Introduction

Eukaryotic mRNAs are co-transcriptionally modified by the addition of a 7-methylguanosine triphosphate moiety at the 5′ end. This modification, referred to as the 5′ cap, protects mRNA from premature degradation by 5′-exonucleases and is necessary for canonical translation initiation. Only specialized decapping enzymes can remove the 7-methylguanosine cap from an mRNA body, thereby exposing it to 5′-to-3′ degradation. Hence, mRNA decapping is an important process, aiding the control of gene expression. Eukaryotic cells produce several decapping enzymes that participate in bulk mRNA decay, mRNA quality control, and specialized degradation pathways.^1−4^ Two major decapping enzymes involved in bulk mRNA degradation are Nudix hydrolase, Dcp2, which is involved in the 5′–3′ degradation pathway and scavenger decapping enzyme, DcpS, which cleaves m^7^G-capped structures released as the products of 3′–5′ mRNA decay.^5,6^ Moreover, other proteins possessing intrinsic decapping activity have been identified and include Nudix family hydrolases (e.g., Nudt3 and Nudt16) and DXO decapping exoribonuclease protein, which is responsible for degrading aberrantly capped RNAs.^7^ Precise control of the activity of those enzymes is crucial to maintain the cell homeostasis.

Some viruses express their own decapping machinery as a part of their strategy to inhibit the cellular translation and promote the synthesis of viral proteins. Till date, four virus-encoded decapping enzymes have been described: D9 and D10 from vaccinia virus (VACV), g5R from African swine fever virus, and L375 Nudix enzyme from mimivirus.^8−11^ All three viruses belong to the family of nucleocytoplasmic large DNA viruses.

**Figure 1:**
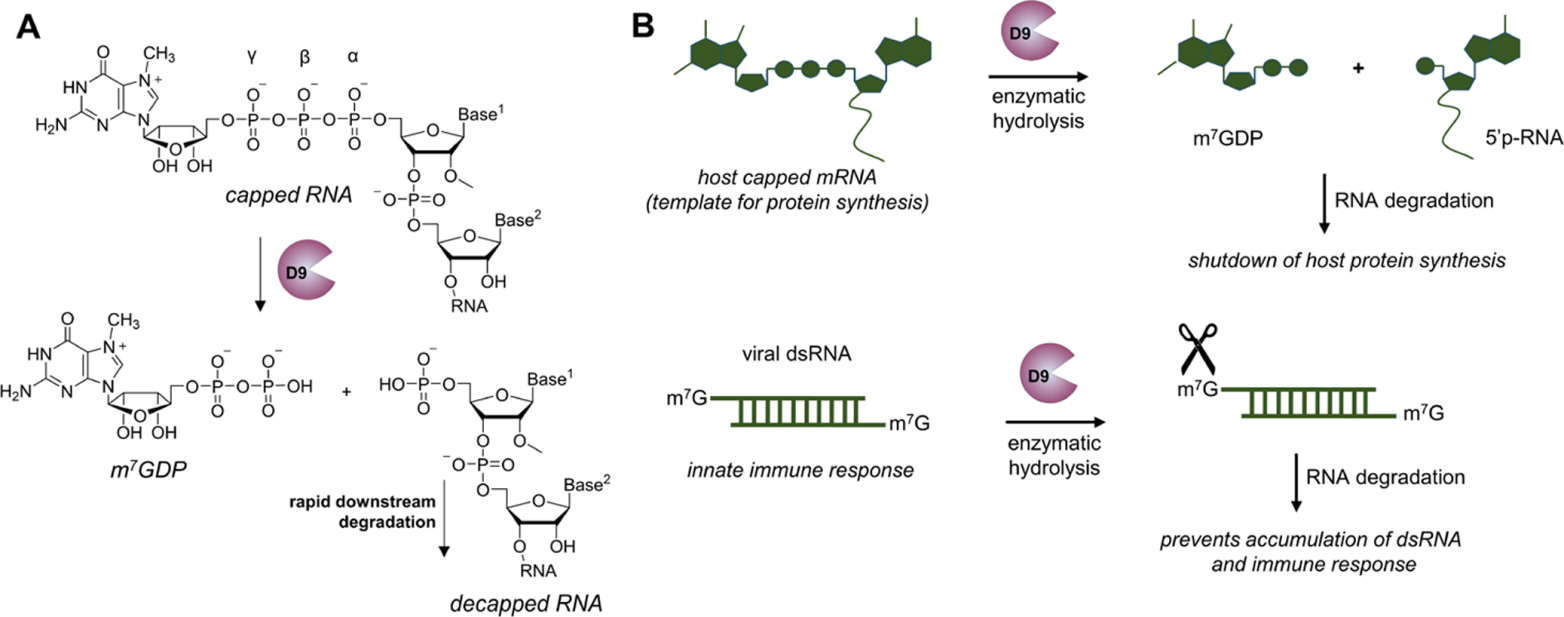
D9 as a viral RNA decapping enzyme. (A) Biochemistry of RNA cleavage and (B) summary of the key D9 functions during viral infection.

The viral decapping enzymes differ greatly in the substrate specificity but share the catalytic motif and the ability to cleave m^7^GpppN-RNAs to release m^7^GDP as a product (Figure 1A). They represent a class of phosphohydrolases that cleave nucleoside diphosphates linked to another moiety X (Nudix). This Nudix motif consists of a 23 amino acid characteristic sequence GX_5_EX_5_[UA]XREX_2_EEXGU, wherein U represents an aliphatic, hydrophobic residue, and X represents any amino acid.^12^ Albeit VACV is not pathogenic to humans, it represents a family of large DNA viruses, including zoonotic viruses, which can jump to humans and/or are pathogenic to livestock and humans. D9 and D10 from VACV are therefore prototype viral decapping enzymes, the studies of which may aid in elucidating how viruses use their decapping machinery to avoid the innate immune response and influence the host translation.

To ensure mRNA stability and translational activity, VACV adds 5′ caps onto viral mRNAs using the virus-encoded capping machinery.^13−15^ Early VACV mRNAs are modified to form either m^7^GpppA_m_ or m^7^GpppG_m_ caps, whereas intermediate and late transcripts carry only m^7^GpppA_m_ caps.^16,17^ D9 is expressed in the early stage of infection and^8^ together with D10, it prevents accumulation of dsRNAs, thereby minimizing cellular antiviral response mediated by PKR and OAS/RNase L.^13,18^ D9 exhibits specificity for N7-methylated cap structures and a preference for longer RNA substrates (>30 nt).^8^ Unmethylated (Gppp-capped) RNAs are not cleaved. The enzyme is strongly inhibited by uncapped RNA, whereas N7-methylated guanine nucleotides such as m^7^GpppG, m^7^GTP, and m^7^GDP are less potent D9 inhibitors. Both D9 and D10 enzymes trigger the degradation of multiple cellular and viral mRNAs to promote the selective translation of viral mRNAs^19^ and reduce the levels of dsRNAs, which are the main trigger of innate immune response (Figure 1B). Interestingly, VACV deficient in either D9 or D10 is more immunogenic due to dsRNA accumulation, replicates more efficiently in cancerous than in healthy cells, and thus acts as effective oncolytic virus.^20^ Significant effort has been made to understand the role of D9 and D10 in VACV replication; nonetheless, the differential functions of these enzymes, their regulation, and interdependence remain elusive.

**Figure 2:**
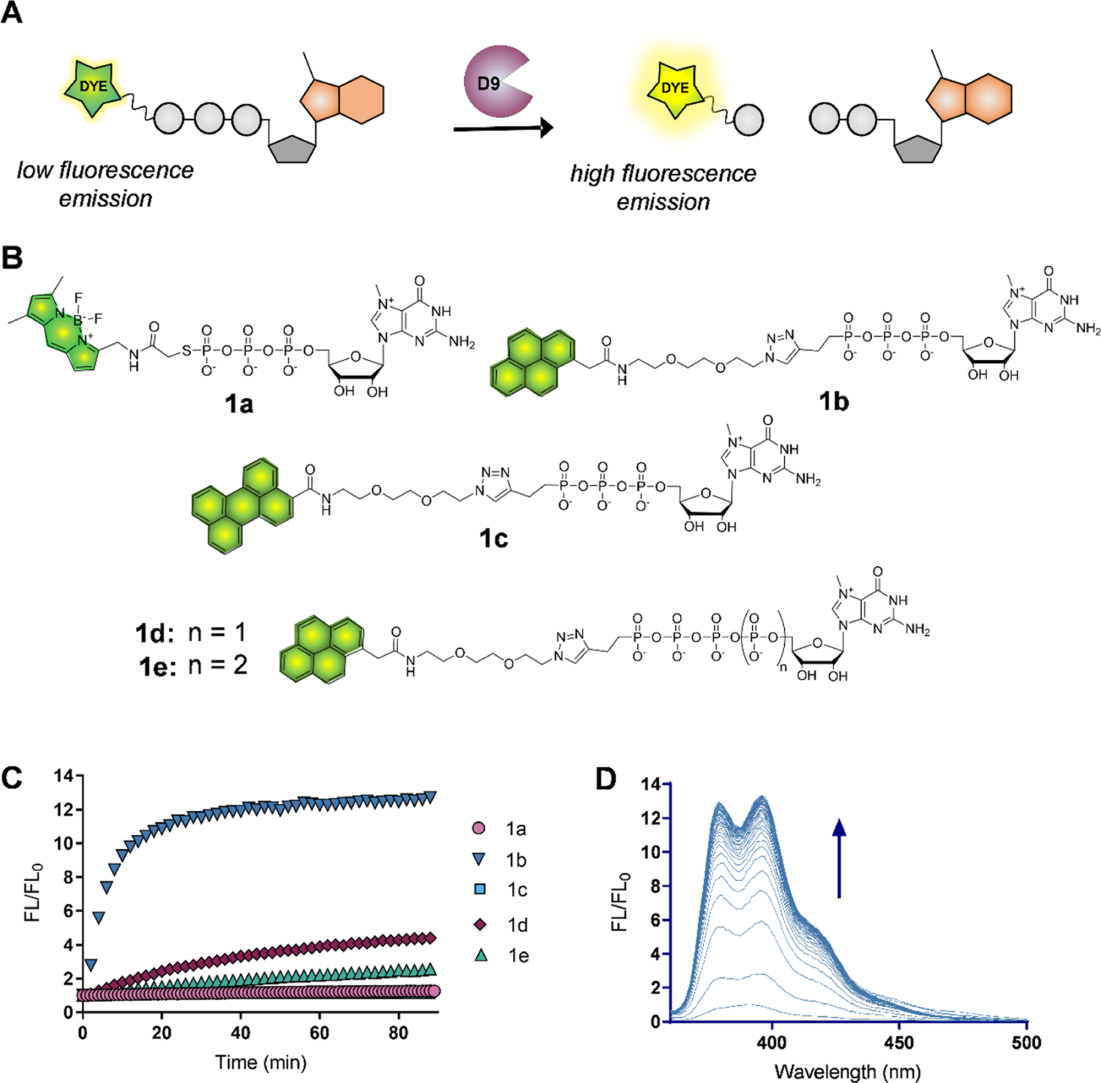
Development of an activity-based assay for D9. (A) Anticipated working mode of fluorescence intensity probes for D9; (B) structures of the evaluated fluorescent probes; and (C) time-dependent fluorescence intensity changes induced by D9 cleavage. Reaction conditions: probe **1a**–**1e** (100 nM), D9 (50 nM) in 10 mM Tris·HCl containing 100 mM KOAc, 2 mM MgCl_2_, and 0.5 mM MnCl_2_, pH 7.5. For each probe, fluorescence changes at emission maximum were monitored (**1a**—exc. 490/em. 512 nm; **1b**, **1d**, and **1e**—345/378 nm, and **1c**—420/489 nm). (D) Time-dependent emission changes for probe **1b** recorded at 1 min interval under conditions given in (C).

Structural and functional studies on cellular decapping enzymes, both *in vitro* and *in vivo*, can be greatly facilitated by the design of specific small-molecule ligands and inhibitors.^21−23^ As such, in this work, we aimed to design molecular tools that could find utility in studying the activity of recombinant D9 and aid discovery of small-molecule inhibitors of this enzyme. Little has been determined about cap-related substrate specificity of D9 or design of specific inhibitors. The so-far-reported inhibition studies relied on isotopically labeled RNA substrates, the use of which entails relatively high cost and low throughput. Here, we sought to design a new simple approach that can be used to study the activity of VACV D9 and discover inhibitors via high-throughput screening (HTS). To this end, we developed fluorescence intensity-based (FLINT) assay with a small-molecule fluorescent probe (Figure 2A). The designed method was adopted to the HTS format to screen an in-house library of nucleotide-derived compounds and a commercially available library of pharmacologically active compounds (LOPAC^1280^, Sigma Aldrich). The identified hits were verified on short RNA substrates and cross-examined against VACV D10 and human decapping enzyme, Dcp1-Dcp2. Finally, one of the most potent inhibitors was crystalized in complex with D9, providing a deeper insight into the inhibition mode.

## Results

### Development of Activity-Based Assay for D9 Enzyme

We first synthesized a set of fluorescently labeled nucleotides as potential fluorogenic substrates for D9. Knowing that D9 specifically cleaves 7-methylguanosine-capped transcripts, all probes were equipped with a 7-methylguanine nucleotide moiety as a recognition element and a fluorescent tag sensitive to m^7^G presence as a cleavage-responsive element (Figure 2A). 7-Methylguanosine has been shown to act as a quencher of electron-rich fluorescent dyes, giving the basis for the development of fluorescent turn-on probes.^24^ To select the most suitable probe in terms of substrate and fluorescent properties, we tested several fluorescent dyes [pyrene (Py), perylene (Pe), and boron dipyrromethene (BODIPY-FL)] and different oligophosphate chain lengths (from tri- to pentaphosphate; Figure 2B). The compounds were subjected to enzymatic cleavage by D9, and emission spectra were recorded over time (Figure 2C,D). In all cases, the gradual increase in fluorescence intensity was observed, consistent with cleavage by D9 enzyme. The greatest changes were observed for probes labeled with pyrene, but the efficiency varied depending on the length of the oligophosphate chain. The system response decreased with the length of the oligophosphate (over 14-, 4-, and 3-fold enhancement of the fluorescence signal for tri-, tetra-, and pentaphosphate, respectively); triphosphates were also the most rapidly cleaved substrates (Figure S1). Therefore, the pyrene-labeled m^7^GTP analogue (**1b**) was selected for further studies as a turn-on probe with the optimal substrate and emission properties. As m^7^GTP-Py is structurally different from natural D9 RNA substrates, we tested whether the hydrolysis mechanism remains similar. The probe was subjected to D9 cleavage, and the reaction progress was monitored using RP-HPLC (Figure S2A,B). Appropriate fractions containing hydrolysis products were collected and identified by mass spectrometry (Figure S2C–F), proving conclusively that D9 enzyme cleaves the probe liberating m^7^GDP and monophosphate of the rest of the molecule, that is, with the same regioselectivity as observed for capped RNA.

**Figure 3:**
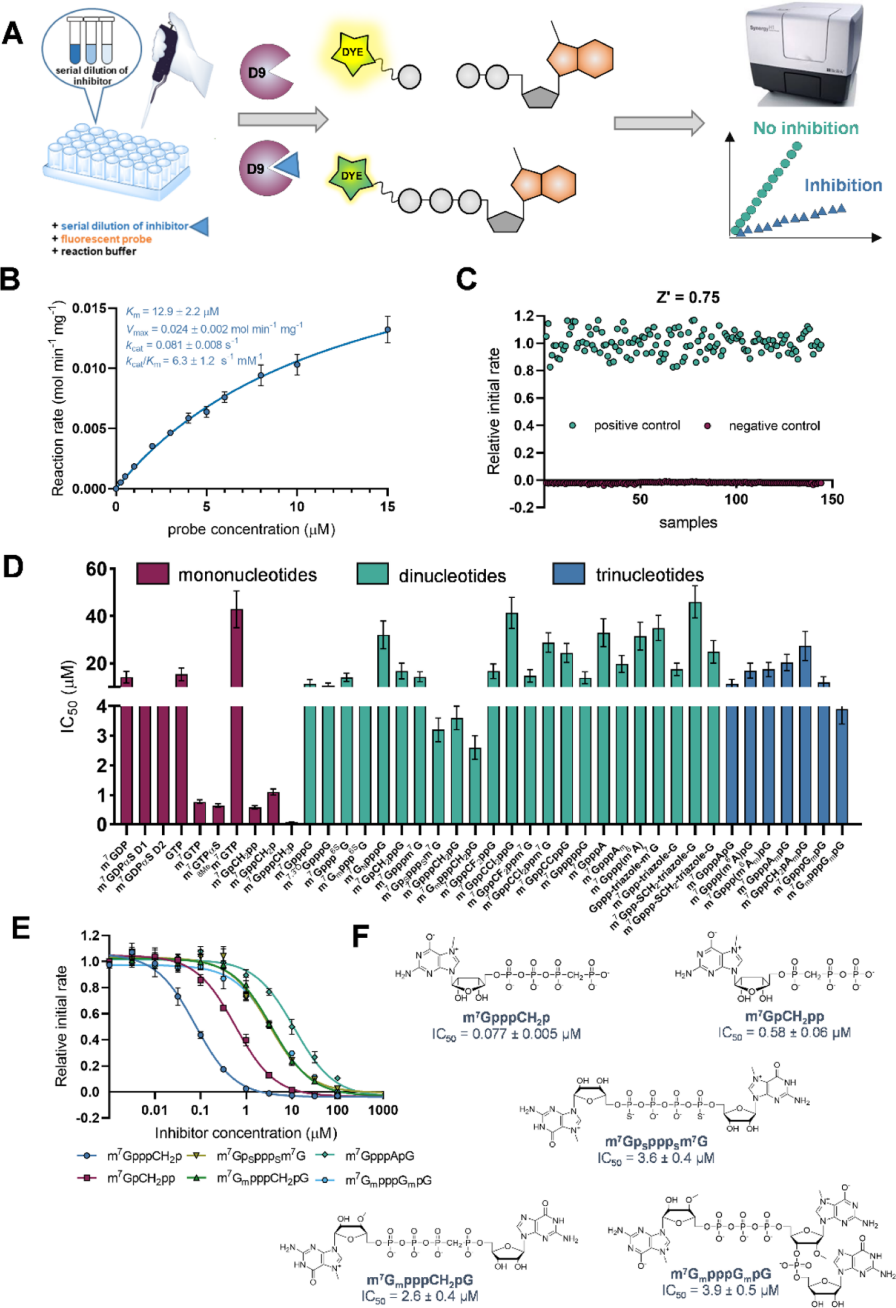
Validation of fluorescence-based assay for D9 activity and evaluation of an in-house library of nucleotide-based compounds. The assay was performed at 30 °C in a 96-well format using 3 μM **1b**, in the presence of the putative inhibitor (half-logarithmic dilution from 100 μM) and 5 nM D9. (A) Application of the fluorescence-based method to monitor D9 activity in the HTS mode; (B) determination of the kinetic parameters for probe **1b** (0–15 μM) in the presence of 5 nM D9. Data shown are means from three independent experiments ±SEM; (C) determination of the *Z*-factor for D9 inhibition assay. Reaction conditions: 3 μM **1b**, 5 nM D9 in the absence (positive control) or presence (negative control) of the 100 μM nucleotide inhibitor. The experiment was carried out in a 96-well plate reader in 10 mM MOPS·HCl, pH 7.0 buffer containing 100 mM KOAc and 2 mM MgCl_2_, 0.3 mM MnCl_2_, 2 mM DTT, and 0.1% BSA at 30 °C; and (D) determined IC_50_ values. Only compounds with IC_50_ < 60 μM are shown. All IC_50_ values, along with compound structures, are shown in Table S1; (E) representative inhibition curves and (F) structures of the most potent compounds from mono-, di-, and trinucleotide groups. Data are shown as mean values from triplicate experiment ±SEM.

Probe **1b** was then characterized in more detail and employed to develop high-throughput screening assay in 96-well plates (Figure 3A). Buffer compositions, concentrations of divalent metal ions, and protein-stabilizing agents were adjusted to optimize the reaction conditions. Manganese (Mn^2+^) ions were essential for efficient enzyme activity (Figure S3A), whereas addition of BSA as a stabilizing protein significantly improved reproducibility (Figure S3B). Initial hydrolysis rates for varying **1b** concentrations were next determined by measuring fluorescence intensity changes as a function of time. Plotting the initial rates as a function of substrate concentrations enabled the determination of Michaelis–Menten kinetic parameters for the probe (*K*_M_, *V*_max_, and *k*_cat_; Figure 3B). Based on those results, we established optimal conditions (Table 1) that ensured sufficient fluorescence signal response, proper reaction kinetics, high protein stability, and good reproducibility. *Z*′ factor^25^ determined under the optimized conditions was 0.75 (Figure 3C), indicating that the quality of the assay is sufficient for the application in high-throughput experiments.

**Table 1:** Optimized Conditions for D9 Hydrolytic Activity Assay Based on Probe **1b**

conditions used in D9 hydrolytic activity experiments	
probe concentration	3 μM
enzyme concentration	5 nM
buffer	10 mM MOPS·HCl, pH 7.0, 100 mM KOAc, containing 2 mM MgCl_2_, 0.3 mM MnCl_2_, 2 mM DTT, and 0.1% BSA
excitation/emission wavelength	345/378 nm
temperature	30 °C
preincubation	15 min, 300 rpm

### Inhibition Studies

To identify the structural preferences for D9 inhibition by substrate analogues, the optimized D9 assay was employed to evaluate an “in-house” library consisting of unmodified mono-, di-, and trinucleotides and their analogues. To assess whether such approach is feasible, we first tested susceptibility of selected nucleotides to D9 cleavage. A set of 15 compounds with different modifications were incubated with D9 for 2 h under assay conditions. Most of the compounds were unsusceptible or did not undergo significant hydrolysis during initial 30 min of the analysis (Figure S4).

Next, the half-maximal inhibitory concentrations (IC_50_) were determined for all nucleotides from the library by observing the degradation of **1b** in the presence of different concentrations of the putative inhibitor (Figure 3D, Table S1). The determined IC_50_ values varied between 0.077 and over 100 μM. The strongest D9 inhibitors (IC_50_ below 1 μM, Figure 3E,F) were identified among mononucleotides containing m^7^G moiety and three or more phosphate groups, indicating that both positively charged 7-methylguanosine and negatively charged phosphate bridge are required for tight interactions with the protein. Dinucleotide cap analogues generally showed weaker inhibitory properties (IC_50_ from 2.6 up to 46 μM) but relatively strong inhibition was observed for several dinucleoside 5′,5′-tetraphosphates, including m^7^G_m_pppCH_2_pG (IC_50_ = 2.6 ± 0.4 μM) and m^7^Gp_S_ppp_S_m^7^G D3 (IC_50_ = 3.6 ± 0.4 μM); the latter has been previously identified as an inhibitor of yeast decapping enzyme SpDcp1/Dcp2.^26^ The most potent compound in the group of trinucleotides was m^7^G_m_pppG_m_pG with IC_50_ value of ∼4 μM. Interestingly, both in the group of dinucleotides and trinucleotides, 2′-*O* methylation of 7-methylguanosine notably increased inhibitory properties, suggesting that this modification might increase binding affinity for D9. At the same time, compounds that were 2′-*O* methylated either at 7-methylguanosine or within the second nucleotide showed the highest susceptibility to hydrolysis by D9 (Figure S4), which might suggest that this is a binding affinity-related effect.

**Figure 4:**
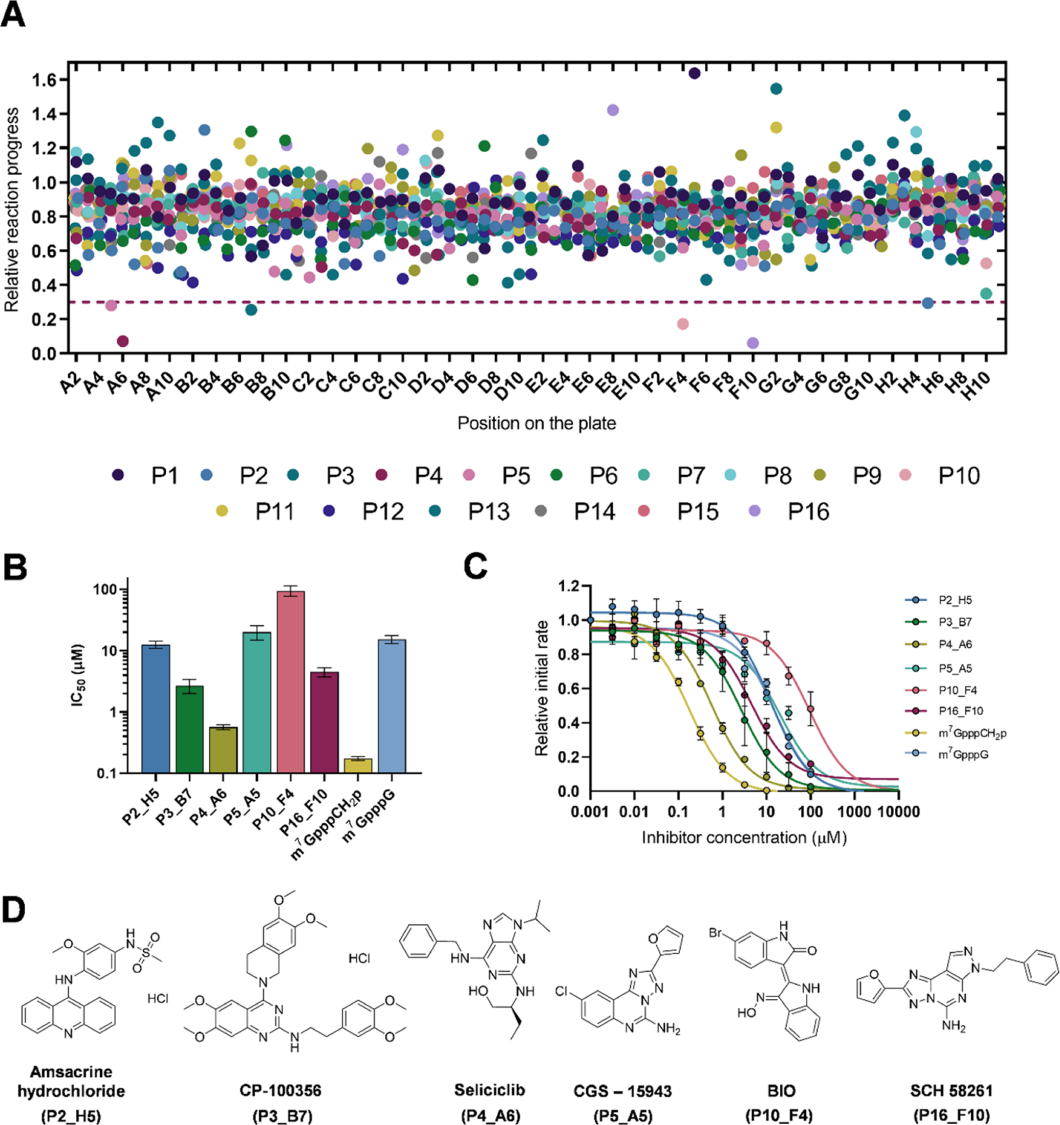
Identification of D9 inhibitors by LOPAC^1280^ library screening. (A) Relative reaction progress of D9-catalyzed (5 nM) hydrolysis of probe **1b** (3 μM) with 1280 compounds from the LOPAC library (30 μM). Data are means from the duplicate experiment; (B) IC_50_ values for six selected hits from the screening experiment (30% cutoff for inhibitor selection). Numerical values are shown in Table 2; (C) inhibition curves for identified inhibitors—one compound had to be tested separately due to its spectroscopic properties. Data are mean values ± SEM from three independent experiments; (D) structures of six most potent hits, and their IC_50_ values are shown in Table 2.

In addition, we screened a commercially available compound library consisting mostly drug-like compounds (LOPAC^1280^, Sigma-Aldrich). Compounds were screened at 30 μM under conditions given in Table 1 (Figure 4A). The relative reaction progress for each compound was calculated by normalizing the initial rate of the reaction in the presence of the tested compound to the initial rate in the absence of any inhibitor. The criterion of the cutoff for inhibitor selection for further evaluation was set to 30% of the maximal initial rate (dotted line), which yielded six potential hits. The hits were then further evaluated, including determination of their IC_50_ values (Figure 4B,C). Three most potent compounds had IC_50_ value below 10 μM, and they included an adenine derivative seliciclib (IC_50_ = 0.57 ± 0.05 μM), quinazolinamine derivative CP-100356 (IC_50_ = 2.7 ± 0.7 μM), and a purine derivative SCH 58261 (4.5 ± 0.8 μM) (Figure 4D).

### Verification of Inhibitory Properties toward D9 for the Identified Hits

**Figure 5:**
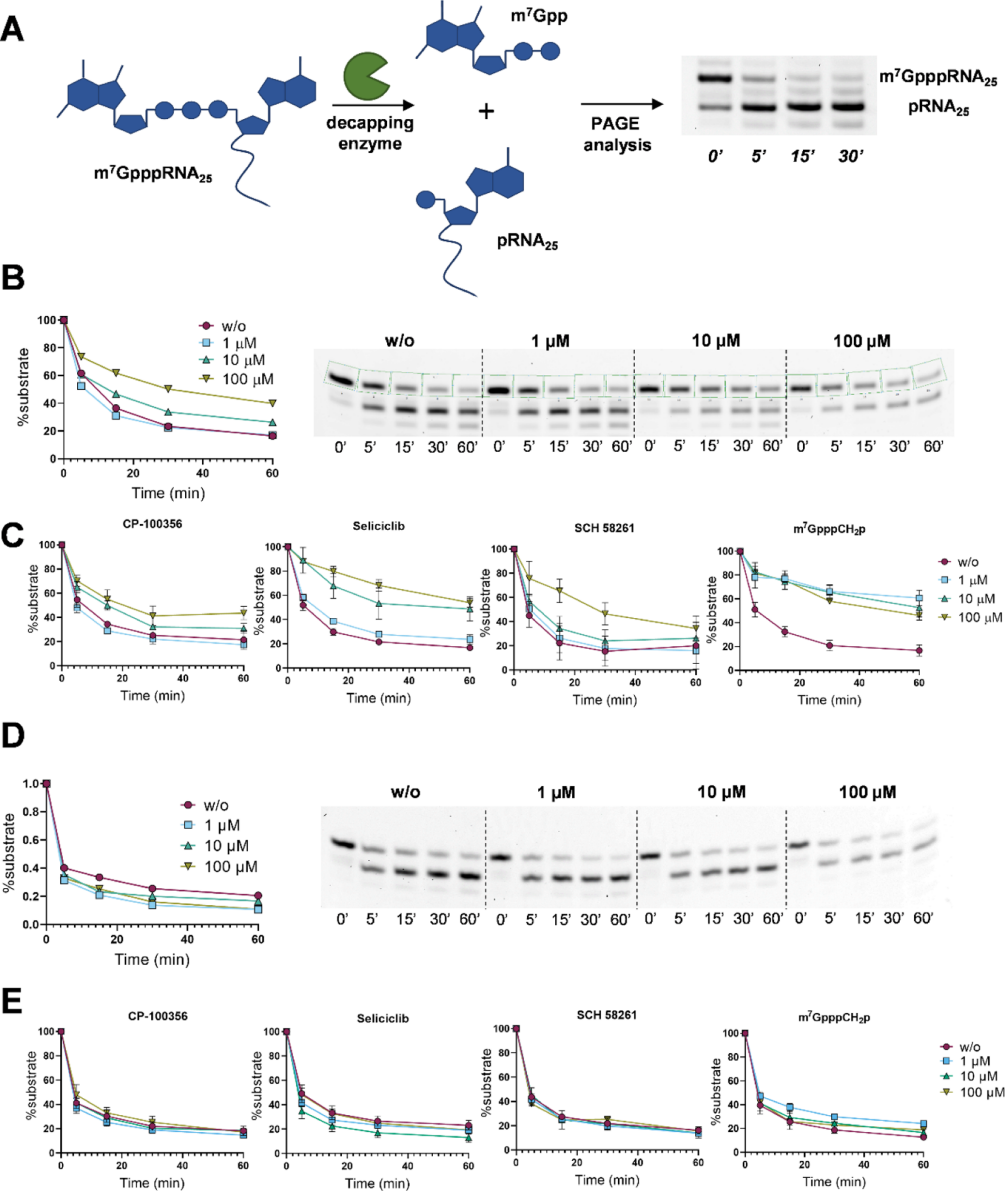
Verification of inhibitory properties and selectivity of the identified hits in a decapping assay on short RNA substrates. (A) Idea of RNA decapping assay based on short m^7^Gppp-RNA substrates (26 nt) and electrophoretic analysis and (B) representative gel (right) from single experiment with compound CP-100356 and densitometric analysis (left) of gel band intensities. m^7^Gppp-capped RNA (20 ng) was incubated for 1 h at 37 °C with D9 (3 nM) without or with the presence of the tested inhibitor at 1, 10, or 100 μM. Samples from different time points were analyzed by polyacrylamide gel electrophoresis (PAGE) and stained with SYBR Gold. The data for all tested compounds are shown in Figure S5; (C) results from triplicate experiments ±SEM for four identified hits confirming their inhibitory properties toward D9 decapping enzyme; and (D,E) analogous experiments with human Dcp1/2 complex (11 nM)—data show no inhibitory properties of tested compounds toward this enzyme. More details are given in the Supporting Information.

To further validate the hits, we checked whether the three most potent inhibitors from the LOPAC library and the best hit from nucleotide-derived library inhibit D9-catalyzed decapping of short capped RNA transcripts. A 26-nt long RNA substrate co-transcriptionally capped with m^7^GpppApG (cap 0 structure)^27^ was subjected to D9-catalyzed hydrolysis in the presence of the evaluated compound at three different concentrations (1, 10, and 100 μM). The reaction progress was analyzed electrophoretically in reference to the reaction performed in the absence of the inhibitor (Figure 5A). The band intensities corresponding to capped and uncapped RNAs were quantified densitometrically, and the decay of the substrate was plotted as a function of time (Figures 5B,C, S5). All compounds showed inhibitory activity toward RNA decapping, and their relative potencies were in good agreement with IC_50_ values determined by the fluorescence assay using probe **1b**. Finally, we tested the specificity of the compounds in the context of VACV D10 and human decapping enzyme Dcp1/2, which are also m^7^GpppRNA hydrolases belonging to the Nudix family. We did not observe any inhibitory activity toward Dcp1/2 for the tested compounds at concentrations up to 100 μM, thereby confirming their selectivity toward D9 (Figures 5D,E, S6). m^7^GpppCH_2_p and CP-100356 showed weak inhibitory activity toward D10 (Figure S7). The most potent drug-like D9 inhibitor identified in this study, seliciclib, showed very good selectivity for D9.

### Crystallographic Structure of Vaccinia Virus D9 with CP-100356

**Figure 6:**
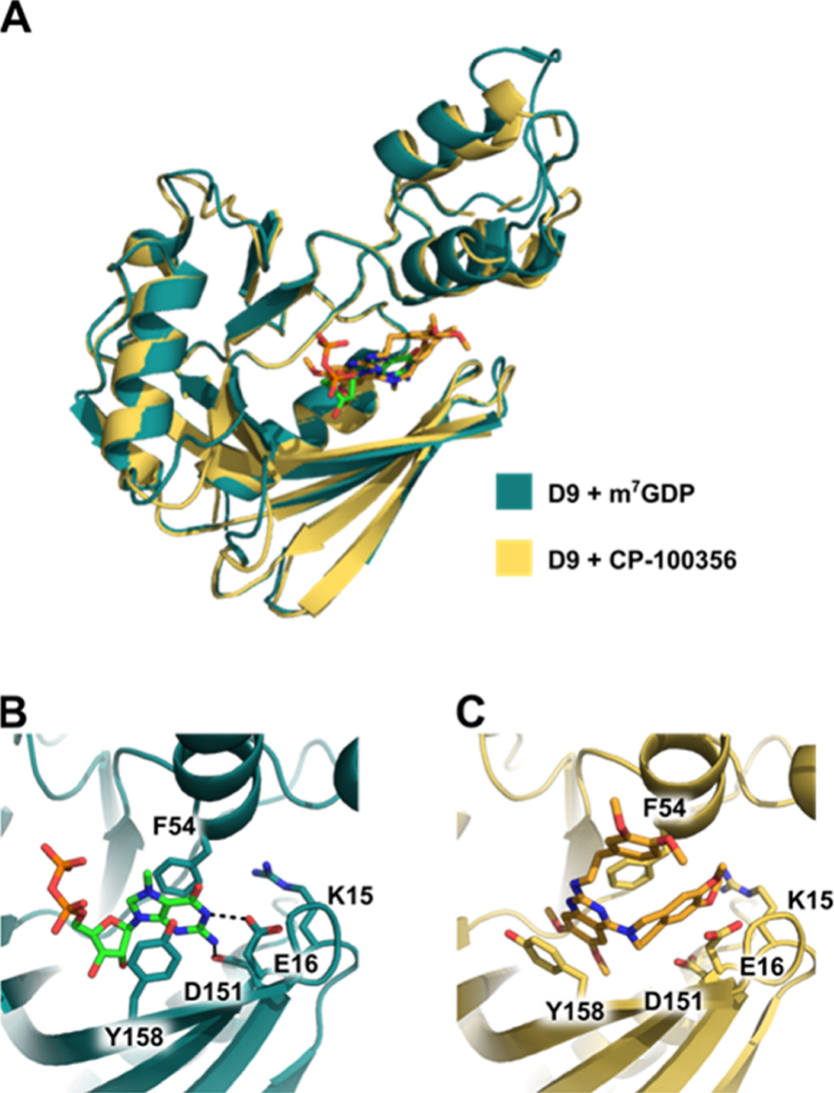
CP-100356 occupies the cap-binding pocket of D9. (A) Alignment of D9 crystal structures obtained by co-crystallizing with m^7^GDP (teal, PDB 7SEZ) or CP-100356 (yellow, PDB 7T7H). The m^7^GDP and CP-100356 molecules are green and orange, respectively. The all-atom RMSD is 2.55 Å. (B) Close-up view showing positioning of the m^7^GDP product in PDB 7SEZ. The methylated guanine base is stacked between conserved aromatic residues F54 and Y158 and hydrogen bonds with E16 and D151. (C) Close-up view showing positioning of the CP-100356 inhibitor in the cap-binding site, sandwiched between F54 and Y158.

To better understand the mechanism of inhibition, we determined the crystal structure of wild-type D9 bound to inhibitor CP-100356 (P3_B7) to 1.78 Å (Figure 6A, Table 3). This crystal form has two copies of D9 in the asymmetric unit (all-atom RMSD 1.811 Å), each bound to 1 molecule of CP-100356. The conformation of CP-100356-bound D9 is nearly identical to a recently published structure of D9 bound to m^7^GDP product in the post-catalytic, inactive conformation (all-atom RMSD 2.55 Å). Our crystal structure reveals that the CP-100356 is positioned in the cap-binding pocket by continuous stacking of the 6,7-dimethoxyquinazoline moiety between aromatic residues F54 and Y158 in a manner similar to m^7^G cap recognition in the product-bound structure, PDB 7SEZ (Figure 6B,C). These conserved residues are essential for efficient cap hydrolysis.^28^ However, in the complex with CP-100356, Y158 is significantly reoriented compared to the position occupied in the complex with m^7^GDP (Figure 6B,C), presumably to allow more efficient π–π stacking interaction with the aromatic quinazoline system of the ligand. Unlike the m^7^G cap in PDB 7SEZ, which is oriented by hydrogen bond base pair mimicry between the guanine base and residues D151 and E16, the CP-100356 inhibitor is not oriented by hydrogen bonding. Rather, the 6,7-dimethoxy-1,2,3,4-tetrahydroisoquinoline moiety extends into a small cavity past D151 and E16 (Figure 6C). It is clear from our crystal structure that CP-100356 inhibits D9 cap hydrolysis by stabilizing the enzyme in an inactive conformation, preventing conserved aromatic residues F54 and Y158 from binding capped mRNA substrate. Attempts to crystallize D9 with the other most potent inhibitors identified from our screen, seliciclib (P4_A6) and SCH 58261 (P16_F10), were unsuccessful. Nonetheless, based on the similarity of these compounds to natural nucleobase moieties, we hypothesize that they likely bind in a similar manner, that is, by stacking between F54 and Y158.

**Table 2:** IC_50_ Values for Inhibitors Identified by HTS Screening

LOPAC ID	compound	IC_50_ (μM)
P2_H5	amsacrine hydrochloride (A 9809)	12.6 ± 1.7
P3_B7	CP-100356 monohydrochloride (PZ0171)	2.7 ± 0.7
P4_A6	seliciclib	0.57 ± 0.05
P5_A5	CGS-15943 (C-199)	20.1 ± 5.2
P10_F4	BIO (B 1686)	95 ± 18
P16_F10	SCH 58261 (S4568)	4.5 ± 0.8
Reference Compounds		
	m^7^GpppCH_2_p	0.18 ± 0.01
	m^7^GpppG	15 ± 2

**Table 3:** Data Collection and Refinement Statistics

	D9 + CP-100356 (PDB: 7T7H)
Data Collection	
space group	*P*2_1_2_1_2_1_
Cell Dimensions	
*a*, *b*, *c* (Å)	56.77, 82.77, 96.31
α, β, γ (deg)	90, 90, 90
resolution (Å)	62.77–1.78 (1.844–1.78)^a^
*R*_merge_	0.04373 (0.9509)
*I*/σ(I)	30.35 (2.29)
completeness (%)	99.79 (97.95)
redundancy	12.9 (12.0)
Refinement	
resolution (Å)	62.77–1.78
no. unique reflections	44,130
*R*_work_/*R*_free_	0.1864/0.2182
No. Atoms	
protein	3294
ligand/ion	84
water	194
*B*-Factors	
protein	48.3
ligand/ion	61.3
water	50.8
RMS Deviations	
bond lengths (Å)	0.019
bond angles (deg)	1.97
Ramachandran (%)	
favored	98
allowed	1.74
outliers	0.26

a Values in parentheses are for the highest resolution shell, and the data set was collected from a single crystal.

## Conclusions

Literature reports that VACV D9 requires RNA body for decapping activity, and that the decapping efficiency increases with the length of the RNA.^8^ Surprisingly, in this work, we discovered a set of minimal substrates (activity probes) for D9 that consist of m^7^GTP molecule connected to a fluorescent tag via the terminal phosphate moiety. The activity probes were evaluated as D9 substrates to select the most efficient and responsive internally quenched probes suitable for high-throughput assay development. Pyrene-labeled m^7^GTP (probe **1b**) had the most favorable properties, both in term of hydrolysis kinetics and fluorescent response upon cleavage. Probably both the m^7^G nucleotide and the pyrene moiety contribute to recognition by D9 because the corresponding unlabeled compounds such m^7^GTP and m^7^GpppG are very poor D9 substrates (as determined by RP HPLC). The probe was used to develop fluorescent HTS assay to screen for D9 inhibitors. The addition of Mn^2+^, beside Mg^2+^ ions, and the presence of BSA were necessary to maintain high protein activity and stability. The optimized assay was characterized by a *Z*′ factor of 0.75, that is, suitable for HTS. The screening experiments were performed on two distinct compound libraries. The first library consisted of nucleotide derivatives, most of which were substrate analogues of varying sizes (mono-, di-, and trinucleotides), and the second was commercially available LOPAC). The nucleotide screening revealed that highly negatively charged 7-methylguanosine mononucleotides are the most potent inhibitors of D9. The LOPAC screening revealed several heteroaromatic compounds as potential hits, which were then further evaluated to show good IC_50_ values and verified inhibitory properties on a longer set of substrates. The most interesting hits identified in the screening were seliciclib and CP-100356. Seliciclib, a 2,6-diaminopurine derivative, was the most potent D9 inhibitor, which combined with its very low inhibitory activity against D10 and hDcp1/2 making it a good candidate for future development. Albeit we were not able to determine the crystal structure of D9 in complex with this inhibitor, its structure suggests that the recognition may be mediated by amino acid-forming nucleic acid-binding sites. CP-100356 was another potent and selective inhibitor of D9 with high potential of future development. The crystal structure of recombinant D9 in complex with CP-100356 was determined, which revealed that the compound targets the m^7^GDP-binding site and forms stacking interactions similar to those observed for 7-methylguanine, suggesting a competitive inhibition mode. This, combined with the observed selectivity of the compound upon cross-examination with VACV D10 and human PNRC2-Dcp1/Dcp2 complex, indicates that the cap-binding site in D9 is distinctive enough from other decapping Nudix family hydrolases to enable the development of small molecules selectively targeting this chemical space.

## Methods

### Expression and Purification of Recombinant Viral D9 and D10 Proteins

Recombinant viral D9 decapping enzyme with additional C-terminal His-tag sequence was expressed, as previously described.^28^ The concentration of the protein was determined spectrophotometrically by assuming ε_280_ = 14,900 M^–1^ cm^–1^. The enzyme was stored at −80 °C in a storage buffer (10 mM MES, pH 7.0, 300 mM NaCl, 1 mM DTT, and 10% glycerol).

Viral D10 decapping enzyme was expressed following a similar protocol, and the detailed procedure will be published elsewhere.

### Expression and Purification of Recombinant Human PNRC2-Dcp1/2 Complex

Human PNRC2-Dcp1/2 decapping complex genes were obtained in the plasmid vector (pETDuet_PNRC2-Dcp1/2). Protein complex (for sequence details, see Figure S8) with His-tag was overexpressed in the *E. coli* BL21(DE3) RIL strain in LB medium with ampicillin (100 μg/mL). Cells were grown to OD_600_ ∼0.5 at 37 °C, then temperature was adjusted to 20 °C. When the culture reached OD_600_ of 0.6–0.7, protein expression was induced by adding isopropyl β-D-1-thiogalactopyranoside (IPTG) to the final concentration of 1 mM, followed by overnight incubation at room temperature with shaking. Cells were then harvested by centrifugation (7000*g*, 4 °C) and lysed by sonication in 50 mM sodium phosphate (pH 7.5), 300 mM NaCl, 10 mM imidazole, 5% glycerol, 1 mM DTT with lysozyme (1 mg/mL), and protease inhibitors (1 mM PMSF, 10.5 μM leupeptin, and 1 μM pepstatin A). The lysate was centrifuged at 40,000*g* for 30 min at 4 °C, and the protein was purified by immobilized metal affinity chromatography on the GE Healthcare HisTrap HP column. The column was washed with 50 mM phosphate (pH 7.5), 1 M NaCl, 30 mM imidazole, and 1 mM DTT, and the PNRC2-Dcp1/2 complex was eluted using 50 mM phosphate buffer (pH 7.5), 300 mM NaCl, 500 mM imidazole, and 1 mM DTT. The supernatant was loaded onto the GE Healthcare HisTrap Heparin HP column and eluted with 50 mM sodium phosphate (pH 7.5), 1 M NaCl, and 1 mM DTT. The last step of purification was size exclusion chromatography on the GE Healthcare HiLoad 16/600 200 pg column. The purified human PNRC2-Dcp1/2 complex was concentrated using 30 kDa Amicon filters and flash frozen in liquid nitrogen. The concentration of the protein was determined spectrophotometrically by assuming ε_280_ = 82,445 M^–1^ cm^–1^. The enzyme was stored at −80 °C in a storage buffer (50 mM Hepes, pH 7.5, 150 mM NaCl, 1 mM DTT, and 20% glycerol).

### Fluorescent Probe Synthesis

All solvents and chemical reagents for non-fluorescent nucleotide synthesis were purchased from Sigma-Aldrich and used without any pre-treatment, unless otherwise indicated. BODIPY FL iodoacetamide was acquired from ThermoFisher Scientific. Pyrene azide and perylene azide were purchased from Lumiprobe. Guanosine and guanosine 5′-monophosphate disodium salts were purchased from Carbosynth. m^7^GTPγS triethylammonium salt was synthesized, as described previously.^29^ Probes **1b**, **1d**, and **1e** were synthesized, as described previously.^24^

Fluorescent nucleotides were purified using analytical or semi-preparative HPLC. Analytical HPLC was performed on Agilent Tech. Series 1200 using the (RP)Supelcosil LC-18-T HPLC column (4.6 × 250 mm, flow rate 1.3 mL/min) with a linear gradient of acetonitrile (0–100% from 0 to 15 min) in 0.05 M ammonium acetate buffer (pH 5.9). UV detection was performed at 260 nm, as well as fluorescent label absorption and fluorescence maximum. Semi-preparative RP HPLC was performed on the same apparatus equipped with the Grace Vision HT C18 HL column (250 cm × 22 mm, 10 μm, flow rate 5.0 mL/min) with a linear gradient of acetonitrile (0–100% from 0 to 120 min) in 0.05 M ammonium acetate buffer (pH 5.9) and UV detection at 260 nm and at the fluorescent tag absorption and emission maximum. The structure and homogeneity of each final product were confirmed by RP HPLC and high-resolution mass spectrometry HRMS (ESI^–^) with a Thermo Scientific LTQ Orbitrap Velos mass spectrometer.

#### m^7^GTPγS-BODIPY-FL (**1a**)

m^7^GTPγS triethylammonium salt (7.5 mg, 8.8 μmol) was mixed with BODIPY-FL iodoacetamide (1.0 mg, 2.4 μmol) in 113 μL of DMSO. The reaction was carried out at room temperature for 1 h and stopped by addition of 0.85 mL of H_2_O. The precipitated fluorescein dye remains were centrifuged. The reaction product was purified using analytical RP HPLC (Method A3). The collected eluate was lyophilized repeatedly to afford 0.5 mg (0.6 μmol) of **1a** as an orange solid. HPLC yield: 58%. Yield after purification: 25%.

HRMS (−)ESI *m*/*z*; found, 841.0976, calcd for C_25_H_31_BF_2_N_8_O_14_P_3_S^–^: 841.3535.

#### m^7^GTPC_4_Pe (**1c**)

Aqueous solution of m^7^Gp_3_C_4_H_5_^30^ triethylammonium salt (1.5 mg, 1.9 μmol) was mixed with a solution of perylene azide (2.0 mg, 4.4 μmol) in DMSO (80 μL), followed by addition of 1 M aqueous solutions of CuSO_4_·5H_2_O (0.8 mg, 3 μmol, 3 μL) and 1 M sodium ascorbate (1.2 mg, 6 μmol, 6 μL). The reaction was carried out at 37 °C for 24 h with shaking (350 rpm) and quenched by addition of Na_2_EDTA (1.8 mg, 4.8 μmol) and water (100 μL). The precipitated perylene dye was separated from the mixture by centrifugation. The reaction product was purified by semi-preparative RP HPLC to give **1c** (0.15 mg, 0.14 μmol) as an ammonium salt. HPLC yield: 81%. Yield after purification: 7.3%.

HRMS (−)ESI *m*/*z*; found, 1024.2221, calcd for C_42_H_45_N_9_O_16_P_3_^–^: 1024.2203.

### Spectroscopic Measurements

Fluorescence emission spectra were recorded using the Agilent Cary Eclipse fluorescence spectrophotometer equipped with the xenon lamp under temperature-controlled conditions. All experiments were performed at 37 °C in the Hellma quartz cuvette (10 × 4 mm) with a sample volume 1000 μL in 10 mM Tris·HCl (pH 7.5), 100 mM KOAc, 2 mM MgCl_2_, 0.5 mM MnCl_2_, and 2 mM DTT. Before each measurement, the buffer was degassed. Emission spectra were registered for all excitation maxima (detailed wavelengths for each probe: **1a**—exc. 490/em. 512 nm; **1b**, **1d**, and **1e**—345/378 nm; and **1c**—420/489 nm).

### Susceptibility to D9 Hydrolysis/Probe Hydrolysis Experiments

The enzymatic activity of D9 was assayed at 30 °C with mixing at 300 rpm in 10 mM Tris·HCl (pH 7.5) containing 100 mM KOAc, 2 mM DTT, 2 mM MgCl_2_, and 0.3 mM MnCl_2_. Reaction mixtures contained 30 μM (nucleotide analogue) or 100 μM (probe **1b**) of the tested compound and 25 nM of the recombinant protein. Enzymatic reaction progress was examined after 5, 15, 30, 45, and 60 min. Aliquots were collected, and the reaction progress was terminated by heat inactivation for 3 min at 95 °C, followed by centrifugation. The samples were analyzed by RP HPLC (Agilent Technologies Series 1200). Appropriate fractions from probe **1b** hydrolysis were collected and identified based on the mass measurements. Mass spectra were recorded on the AB Sciex API 3200 spectrometer.

### Point Fluorescence Measurements—General Information

Point fluorescence measurements were performed using the BioTek Synergy H1 microplate reader. Before each measurement, the reaction buffer was degassed. Experiments were performed in the Greiner 96-well, black, non-binding plates at 30 °C. Before the measurement, the plate was preincubated for 15 min at 30 °C with mixing (300 rpm). The point fluorescence was registered with 1 min interval and detection at 345 nm excitation wavelength and 378 nm emission wavelength.

### Kinetic Parameter Determination

To determine the kinetic parameters of enzymatic hydrolysis of probe **1b** by D9 enzyme, point fluorescence measurements were performed. Each well of the plate contained an appropriate buffer (10 mM MOPS·HCl, pH 7.0, 100 mM KOAc, containing 2 mM MgCl_2_, 0.3 mM MnCl_2_, and 2 mM DTT) with addition of 0.1% BSA, a substrate (probe **1b** at a concentration range 0–15 μM) and an enzyme (5 nM of D9) to a final volume of 150 μL. The measurement was carried out on until full saturation, which allowed us to convert the initial rates from au/min to mol/min/mg. The initial rates were calculated by fitting a linear curve to the first 10 points (10 min) of the hydrolysis reaction. To calculate the kinetic parameters, the Michaelis–Menten model was applied with the following formula fitted to the experimental data using GraphPad Prism software
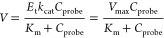
where *V* is the initial rate of the reaction, *E*_t_ is the concentration of enzyme sites, *k*_cat_ is the turnover number indicating the catalytic efficiency, *C*_probe_ is the concentration of the probe **1b**, *K*_m_ is the Michaelis–Menten constant, and *V*_max_ is the maximum enzyme velocity.

### *Z*′ Factor Determination

To determine the *Z*′ factor of the method, point fluorescence measurements were performed. The positive control contained a mixture of probe **1b** (3 μM) and enzyme D9 (5 nM) in 10 mM MOPS·HCl, pH 7.0, 100 mM KOAc, containing 2 mM MgCl_2_, 0.3 mM MnCl_2_, and 2 mM DTT. The negative control additionally included a nucleotide inhibitor (30 μM of m^7^GpppCH_2_p^31^) to fully hinder the D9 activity. The value of *Z′* parameter was calculated as follows
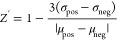
where σ_pos_ and σ_neg_ are the standard deviations and μ_pos_ and μ_neg_ are the mean values of the positive and negative controls, respectively.

### High-Throughput Screening of the LOPAC^1280^ Library

To screen the LOPAC^1280^ Library, point fluorescence measurements were performed. Each plate well contained an appropriate buffer (10 mM MOPS·HCl, pH 7.0, 100 mM KOAc, containing 2 mM MgCl_2_, 0.3 mM MnCl_2_, and 2 mM DTT) with addition of 0.1% BSA, a fluorescent substrate (3 μM of probe **1b**), an inhibitor (30 μM of each LOPAC compound), and an enzyme (5 nM of D9) to a final volume of 150 μL. To determine the relative reaction progress, the ratio of the initial reaction rate with inhibitor to the initial rate without inhibitor was calculated. Nine out of the 1280 compounds showed interference with pyrene fluorescence and were tested separately with different settings of fluorescence read-out (different gains).

### IC_50_ Parameter Evaluation

Experiments to determine IC_50_ parameters were performed as point fluorescence measurements. Each well contained an appropriate buffer (10 mM MOPS·HCl, pH 7.0, 100 mM KOAc, containing 2 mM MgCl_2_, 0.3 mM MnCl_2_, and 2 mM DTT) with addition of 0.1% BSA, a fluorescent substrate (3 μM of probe **1b**), an inhibitor (in half-logarithmic dilution, *c*_inh_ ∈ 0, 100 μM), and an enzyme (5 nM of D9) to a final volume of 150 μL. To determine the IC_50_ values, a three parameter dose–response formula with standard hill slope from GraphPad Prism software was fitted
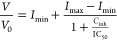
where *V*/*V*_0_ is the relative initial rate of the reaction, *I*_min_ and *I*_max_ are bottom and top plateaus, and *C*_inh_ is the concentration of the inhibitor.

All tested compounds come from LOPAC^1280^ (Table 2) or our in-house library of cap analogues (determined IC_50_ values with compound’s structures and synthesis references are available in Table S1).

### RNA Synthesis

Short RNA sequences were synthesized by in vitro transcription (IVT) using T7 class II promoter Φ2.5 initiated by ATP (TAATACGACTCACTATTA).^32^ The typical transcription reaction (200 μL) was carried out for 4 h at 37 °C and contained 5 mM each of UTP/GTP/CTP (Thermo Fisher Scientific), 1.25 mM ATP (Thermo Fisher Scientific), and 6.5 mM of trinucleotide cap analogue (m^7^GpppApG), RNA Polymerase Buffer (Thermo Fisher Scientific), 1 μM of annealed oligonucleotides as DNA template (CAGTAATACGACTCACTATTAGGGAAGCGGGCATGCGGCCAGCCATAGCCGATCA and TGATCGGCTATGGCTGGCCGCATGCCCGCTTCCCTAATAGTGAGTCGTATTACTG), 1 U/μL RiboLock RNase inhibitor (Thermo Fisher Scientific), 20 mM MgCl_2_, and T7 RNA polymerase (20 U/μL Thermo Fisher Scientific). Uncapped transcripts (IVT) were obtained as mentioned above but in the presence of 5 mM ATP and without addition of cap analogue. After 4 h incubation, 7 U of DNase I (Thermo Fisher Scientific) was added, and the mixture was incubated for another 30 min at 37 °C. The reaction mixture was stopped by the addition of equimolar amounts of Na_2_EDTA water solution (EDTA to Mg^2+^) and RNA phenol–chloroform extraction, followed by RNA precipitation (3 M NaOAc pH 5.2, ethanol). The crude RNA sample was next purified using the HPLC and Phenomenex Clarity 3 μM Oligo-RP column (150 × 4.6 mm) (the method is given below) and precipitated as before. To obtain a homogeneous RNAs at 3′ end, appropriate sample underwent trimming by DNAzyme 10–23 (TGATCGGCTAGGCTAGCTACAACGAGGCTGGCCGC). The reaction mixture contains equal molar concentrations of RNA substrates and DNAzyme in 50 mM Tris (pH 8.0) and 50 mM MgCl_2_ for 1 h at 37 °C. The reaction was then purified using HPLC, and the desired product was precipitated with ethanol as mentioned above. This procedure allows us to obtain a 3′ homogeneous 25-nt (uncapped) or 26-nt (capped) transcripts. The quality of the obtained short RNAs was checked on 15% acrylamide/7 M urea/TBE gels with SYBR Gold (Invitrogen) staining, and the concentration was determined spectrophotometrically.

HPLC method: A: 100 mM TEAAc, B: 200 mM TEAAc/ACN; 10–30% of eluent B in 30 min.

### RNA Decapping Assay

20 ng of 26-nt capped RNA transcript was incubated with an appropriate decapping enzyme (3 nM D9 or 11 nM Dcp1/2 or 200 nM D10) in an appropriate buffer with addition of 0.1% BSA at 37 °C. After being indicated, times reactions were terminated by adding equal volume of the loading dye (4.5 M urea, 50% formamide, 20 mM EDTA, 0.03% bromophenol blue, and 0.03% xylene cyanol) and flash freezing. Samples were then resolved by PAGE on denaturing 15% acrylamide/7 M urea/TBE gel and were stained with SYBR Gold (Invitrogen) and visualized using a Typhoon FLA 9500 (GE Healthcare). The band intensities corresponding to capped and uncapped RNAs were quantified densitometrically using 1D Gel Image Analysis Software TotalLab CLIQS.

Buffer used in D9 experiments: 10 mM MOPS·KOH (pH = 7.0), 100 mM KOAc, 2 mM MgCl_2_, 0.3 mM MnCl_2_, and 2 mM DTT.

Buffer used in Dcp2 experiments: 50 mM Tris·HCl pH = 8.0, 50 mM NH_4_Cl, 0.01% Igepal, 5 mM MgCl_2_, 2 mM MnCl_2_, and 1 mM DTT.

Buffer used in D10 experiments: 10 mM MOPS·KOH pH = 7.0, 100 mM KOAc, 2 mM MgCl_2_, 0.3 mM MnCl_2_, and 2 mM DTT.

### X-ray Crystallography

The CP-100356 inhibitor was dissolved in DMSO at a concentration of 5 mg/mL. The protein–inhibitor complex was prepared by mixing D9 and CP-100356 in crystallization buffer containing 20 mM MgCl_2_ to a final concentration of 10 mg/mL D9 and 3 mM cap analogue substrates and incubated at room temperature for 30 min. The protein–substrate complex was mixed with well solution at a 1:1 ratio, and crystals were grown by hanging drop vapor diffusion at room temperature. D9:CP-100356 well solution contained 20% PEG 3350 and 100 mM NaF. Crystals were flash frozen in liquid nitrogen using a cryoprotectant consisting of well solution with 25% glycerol. All data sets were collected on beamline 8.3.1 at the Advanced Light Source at 100 K and wavelength 1.11583 Å using the Pilatus3 S 6 M detector and indexed, integrated, and scaled using XDS,^33^ Pointless and Aimless^34^ via automated beamline software ELVES.^35^ Phasing was carried out by molecular replacement with the previously solved D9 structure in the post-catalytic conformation, 7SEZ.^28^ The structure was then iteratively refined in PHENIX^36^ and manually adjusted in COOT.^37^

## Supporting Information Available

Detailed results of inhibitory studies obtained from FLINT assay, kinetic and hydrolysis experiments, HTS assay optimization, susceptibility to D9 hydrolysis, in vitro decapping assay with PAGE gels and sequences of the used proteins, and HPLC profiles and HRMS spectra for newly synthesized compounds (PDF)

## References

[ref1] Łabno A.; Tomecki R.; Dziembowski A. Cytoplasmic RNA decay pathways - Enzymes and mechanisms. Biochim. Biophys. Acta Mol. Cell Res. 2016, 1863, 3125–3147. 10.1016/j.bbamcr.2016.09.023. 27713097

[ref2] Lykke-Andersen S.; Jensen T. H. Nonsense-mediated mRNA decay: an intricate machinery that shapes transcriptomes. Nat. Rev. Mol. Cell Biol. 2015, 16, 665–677. 10.1038/nrm4063. 26397022

[ref3] Barreau C.; Paillard L.; Osborne H. B. AU-rich elements and associated factors: are there unifying principles?. Nucleic Acids Res. 2005, 33, 7138–7150. 10.1093/nar/gki1012. 16391004 PMC1325018

[ref4] Jonas S.; Izaurralde E. Towards a molecular understanding of microRNA-mediated gene silencing. Nat. Rev. Genet. 2015, 16, 421–433. 10.1038/nrg3965. 26077373

[ref5] Liu H.; Rodgers N. D.; Jiao X.; Kiledjian M. The scavenger mRNA decapping enzyme DcpS is a member of the HIT family of pyrophosphatases. EMBO J. 2002, 21, 4699–4708. 10.1093/emboj/cdf448. 12198172 PMC126188

[ref6] van Dijk E.; Cougot N.; Meyer S.; Babajko S.; Wahle E.; Seraphin B. Human Dcp2: a catalytically active mRNA decapping enzyme located in specific cytoplasmic structures. EMBO J. 2002, 21, 6915–6924. 10.1093/emboj/cdf678. 12486012 PMC139098

[ref7] Grudzien-Nogalska E.; Kiledjian M. New insights into decapping enzymes and selective mRNA decay. Wiley Interdiscip. Rev.-Rna 2017, 8, 11 10.1002/wrna.1379. PMC5179306 27425147

[ref8] Parrish S.; Moss B. Characterization of a second vaccinia virus mRNA-decapping enzyme conserved in poxviruses. J. Virol. 2007, 81, 12973–12978. 10.1128/jvi.01668-07. 17881455 PMC2169080

[ref9] Parrish S.; Resch W.; Moss B. Vaccinia virus D10 protein has mRNA decapping activity, providing a mechanism for control of host and viral gene expression. Proc. Natl. Acad. Sci. U.S.A. 2007, 104, 2139–2144. 10.1073/pnas.0611685104. 17283339 PMC1793903

[ref10] Parrish S.; Hurchalla M.; Liu S.-W.; Moss B. The African swine fever virus g5R protein possesses mRNA decapping activity. Virology 2009, 393, 177–182. 10.1016/j.virol.2009.07.026. 19695654 PMC3392020

[ref11] Kago G.; Parrish S. The Mimivirus L375 Nudix enzyme hydrolyzes the 5’ mRNA cap. PLoS One 2021, 16, e0245820 10.1371/journal.pone.0245820. 34582446 PMC8478210

[ref12] McLennan A. G. The Nudix hydrolase superfamily. Cell. Mol. Life Sci. 2006, 63, 123–143. 10.1007/s00018-005-5386-7. 16378245 PMC11136074

[ref13] Liu S.-W.; Katsafanas G. C.; Liu R.; Wyatt L. S.; Moss B. Poxvirus Decapping Enzymes Enhance Virulence by Preventing the Accumulation of dsRNA and the Induction of Innate Antiviral Responses. Cell Host Microbe 2015, 17, 320–331. 10.1016/j.chom.2015.02.002. 25766293 PMC4359750

[ref14] Dhungel P.; Cantu F. M.; Molina J. A.; Yang Z. Vaccinia Virus as a Master of Host Shutoff Induction: Targeting Processes of the Central Dogma and Beyond. Pathogens 2020, 9, 400 10.3390/pathogens9050400. PMC7281567 32455727

[ref15] Moss B. Poxviridae; Wolters Kluwer Health/Lippincott Williams & Wilkins: Philadelphia, 2013.

[ref16] McLennan A. G. Decapitation: poxvirus makes RNA lose its head. Trends Biochem. Sci. 2007, 32, 297–299. 10.1016/j.tibs.2007.05.001. 17498957

[ref17] Baldick C. J.; Moss B. Characterization and temporal regulation of messenger-RNAs encoded by Vaccinia Virus intermediate-stage genes. J. Virol. 1993, 67, 3515–3527. 10.1128/jvi.67.6.3515-3527.1993. 8098779 PMC237698

[ref18] Silverman R. H. Viral encounters with 2’,5’-oligoadenylate synthetase and RNase L during the interferon antiviral response. J. Virol. 2007, 81, 12720–12729. 10.1128/jvi.01471-07. 17804500 PMC2169107

[ref19] Cantu F.; Cao S.; Hernandez C.; Dhungel P.; Spradlin J.; Yang Z. Poxvirus-encoded decapping enzymes promote selective translation of viral mRNAs. PLoS Pathog. 2020, 16, e1008926 10.1371/journal.ppat.1008926. 33031446 PMC7575113

[ref20] Burgess H. M.; Pourchet A.; Hajdu C. H.; Chiriboga L.; Frey A. B.; Mohr I. Targeting Poxvirus Decapping Enzymes and mRNA Decay to Generate an Effective Oncolytic Virus. Mol. Ther.--Oncolytics 2018, 8, 71–81. 10.1016/j.omto.2018.01.001. 29888320 PMC5991893

[ref21] Mugridge J. S.; Tibble R. W.; Ziemniak M.; Jemielity J.; Gross J. D. Structure of the activated Edc1-Dcp1-Dcp2-Edc3 mRNA decapping complex with substrate analog poised for catalysis. Nat. Commun. 2018, 9, 10 10.1038/s41467-018-03536-x. 29559651 PMC5861098

[ref22] Luo Y.; Schofield J. A.; Na Z.; Hann T.; Simon M. D.; Slavoff S. A. Discovery of cellular substrates of human RNA-decapping enzyme DCP2 using a stapled bicyclic peptide inhibitor. Cell Chem. Biol. 2021, 28, 463–474. e7 10.1016/j.chembiol.2020.12.003. 33357462 PMC8052284

[ref23] Souliere M. F.; Perreault J.-P.; Bisaillon M. Insights into the molecular determinants involved in cap recognition by the vaccinia virus D10 decapping enzyme. Nucleic Acids Res. 2010, 38, 7599–7610. 10.1093/nar/gkq628. 20639534 PMC2995054

[ref24] Kasprzyk R.; Starek B. J.; Ciechanowicz S.; Kubacka D.; Kowalska J.; Jemielity J. Fluorescent Turn-On Probes for the Development of Binding and Hydrolytic Activity Assays for mRNA Cap-Recognizing Proteins. Chem.—Eur. J. 2019, 25, 6728–6740. 10.1002/chem.201900051. 30801798

[ref25] Zhang J.-H.; Chung T. D. Y.; Oldenburg K. R. A simple statistical parameter for use in evaluation and validation of high throughput screening assays. J. Biomol. Screening 1999, 4, 67–73. 10.1177/108705719900400206. 10838414

[ref26] Ziemniak M.; Mugridge J. S.; Kowalska J.; Rhoads R. E.; Gross J. D.; Jemielity J. Two-headed tetraphosphate cap analogs are inhibitors of the Dcp1/2 RNA decapping complex. Rna 2016, 22, 518–529. 10.1261/rna.055152.115. 26826132 PMC4793208

[ref27] Sikorski P. J.; Warminski M.; Kubacka D.; Ratajczak T.; Nowis D.; Kowalska J.; Jemielity J. The identity and methylation status of the first transcribed nucleotide in eukaryotic mRNA 5’ cap modulates protein expression in living cells. Nucleic Acids Res. 2020, 48, 1607–1626. 10.1093/nar/gkaa032. 31984425 PMC7038993

[ref28] Peters J. K.; Tibble R. W.; Warminski M.; Jemielity J.; Gross J. D. Structure of the poxvirus decapping enzyme D9 reveals its mechanism of cap recognition and catalysis. Structure 2022, 30, 721–732. 10.1016/j.str.2022.02.012. 35290794 PMC9081138

[ref29] Kowalska J.; Lewdorowicz M.; Darzynkiewicz E.; Jemielity J. A simple and rapid synthesis of nucleotide analogues containing a phosphorothioate moiety at the terminal position of the phosphate chain. Tetrahedron Lett. 2007, 48, 5475–5479. 10.1016/j.tetlet.2007.05.170.

[ref30] Walczak S.; Nowicka A.; Kubacka D.; Fac K.; Wanat P.; Mroczek S.; Kowalska J.; Jemielity J. A novel route for preparing 5 ’ cap mimics and capped RNAs: phosphate-modified cap analogues obtained via click chemistry. Chem. Sci. 2017, 8, 260–267. 10.1039/c6sc02437h. 28451173 PMC5355871

[ref31] Jemielity J.; Pietrowska-Borek M.; Starzynska E.; Kowalska J.; Stolarski R.; Guranowski A.; Darzynkiewicz E. Synthesis and enzymatic characterization of methylene analogs of adenosine 5’-tetraphosphate (P4A). Nucleosides, Nucleotides Nucleic Acids 2005, 24, 589–593. 10.1081/NCN-200061911. 16247994

[ref32] Coleman T. M.; Wang G. C.; Huang F. Q. Superior 5’ homogeneity of RNA from ATP-initiated transcription under the T7 phi 2.5 promoter. Nucleic Acids Res. 2004, 32, 14e 10.1093/nar/gnh007. PMC373309 14744982

[ref33] Kabsch W. XDS. Acta Crystallogr., Sect. D: Biol. Crystallogr. 2010, 66, 125–132. 10.1107/s0907444909047337. 20124692 PMC2815665

[ref34] Winn M. D.; Ballard C. C.; Cowtan K. D.; Dodson E. J.; Emsley P.; Evans P. R.; Keegan R. M.; Krissinel E. B.; Leslie A. G. W.; McCoy A.; et al. Overview of the CCP4 suite and current developments. Acta Crystallogr., Sect. D: Struct. Biol. 2011, 67, 235–242. 10.1107/s0907444910045749. PMC3069738 21460441

[ref35] Holton J.; Alber T. Automated protein crystal structure determination using ELVES. Proc. Natl. Acad. Sci. U.S.A. 2004, 101, 1537–1542. 10.1073/pnas.0306241101. 14752198 PMC341770

[ref36] Adams P. D.; Afonine P. V.; Bunkóczi G.; Chen V. B.; Davis I. W.; Echols N.; Headd J. J.; Hung L.-W.; Kapral G. J.; Grosse-Kunstleve R. W.; et al. PHENIX: a comprehensive Python-based system for macromolecular structure solution. Acta Crystallogr., Sect. D: Struct. Biol. 2010, 66, 213–221. 10.1107/s0907444909052925. PMC2815670 20124702

[ref37] Emsley P.; Lohkamp B.; Scott W. G.; Cowtan K. Features and development of Coot. Acta Crystallogr., Sect. D: Biol. Crystallogr. 2010, 66, 486–501. 10.1107/s0907444910007493. 20383002 PMC2852313

