# Supplementary material for: Fluorescence-Based Activity Screening Assay Reveals Small Molecule Inhibitors of Vaccinia Virus mRNA Decapping Enzyme D9
Source: ACS Chem Biol. 2022 May 16;17(6):1460–71. doi: 10.1021/acschembio.2c00049 (PMC9207806; doi:10.1021/acschembio.2c00049)
Supplement: Supplementary file 1 — cb2c00049_si_001.pdf [file cb2c00049_si_001.pdf]

Supporting Information

A fluorescence-based activity screening assay reveals small molecule inhibitors of Vaccinia Virus mRNA decapping enzyme D9

Marcelina Bednarczyk,[1,2] Jessica K. Peters,[3] Renata Kasprzyk,[2] Jagoda Starek,[1] Marcin Warminski,[1] Tomasz Spiewla,[1] Jeffrey S. Mugridge,[3,4] John D. Gross[3], Jacek Jemielity,*[,2] Joanna Kowalska,*[,1]

[1] Division of Biophysics, Institute of Experimental Physics, Faculty of Physics, University of Warsaw, Pasteura 5, 02-093 Warsaw, Poland

[2] Centre of New Technologies, University of Warsaw, Banacha 2C, 02-097 Warsaw, Poland

[3] Department of Pharmaceutical Chemistry, University of California, San Francisco, CA 94158, USA

[4] Department of Chemistry & Biochemistry, University of Delaware, Newark, DE 19716, USA

*corresponding authors: j.jemielity@cent.uw.edu.pl, jkowalska@fuw.edu.pl

Table of contents

1. **Supporting Tables** **S2**
2. **Supporting Figures** **S6**
3. **Raw data: HPLC and HRMS profiles** **S19**
4. **Supporting References** **S21**

1. Supporting tables.

Table S1. IC_{50} values of all tested nucleotide-derived compounds obtained from FLINT HTS assay. Data shown are means of 3 independent experiments ± SEM.

No	Compound	Structure	IC_{50} (µM)	Ref to synthesis
	mononucleotides			
**1.**	m^{7}GMP	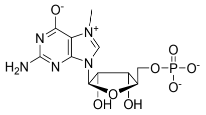	>>100	[1]
**2.**	m^{7}GDP	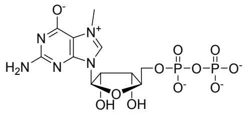	14.2 ± 2.4	[1]
**3.**	m^{7}GDPαS D1	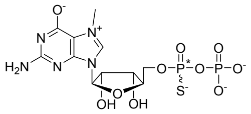	8.3 ± 1.7	[2]
**4.**	m^{7}GDPαS D2	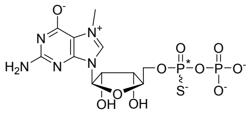	7.6 ± 1.2	[2]
**5.**	^{8Me}m^{7}GDP	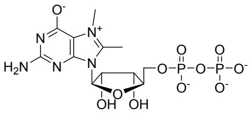	n.d (>>100)	unpublished
**6.**	m^{7}GTP	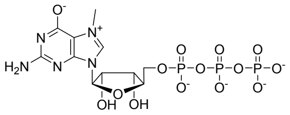	0.77 ± 0.06	[1]
**7.**	GTP	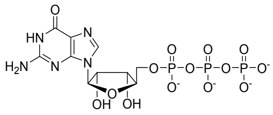	15.4 ± 2.7	commercially available
**8.**	m^{7}GTPαS	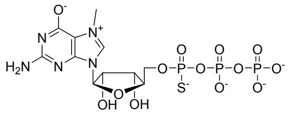	0.64 ± 0.06	[3]
**9.**	^{8Me}m^{7}GTP	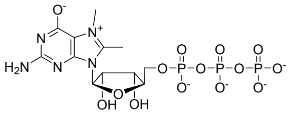	42.9 ± 7.8	unpublished
**10.**	m^{7}GpCH$_2$pp	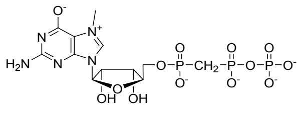	0.58 ± 0.06	[4]
**11.**	m^{7}GppCH$_2$p	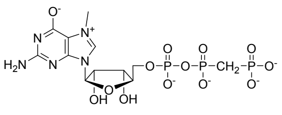	1.1 ± 0.1	[5]

**12.**	m^{7}GpppCH$_2$p	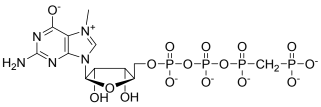	0.077 ± 0.008	[5]
	dinucleotides			
**13.**	m^{7}GpppG	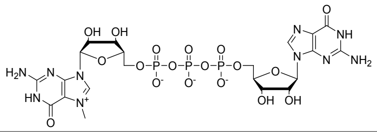	11.3 ± 1.9	[6]
**14.**	GpppG	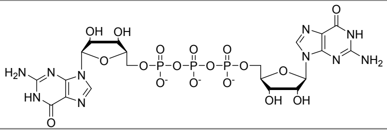	>>100	[7]
**15.**	m$_2$$^{7,3'O}$GpppG	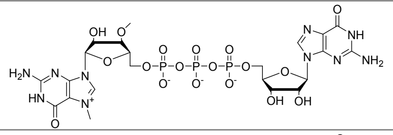	10.6 ± 1.1	[8]
**16.**	m^{7}Gppp^{6S}G	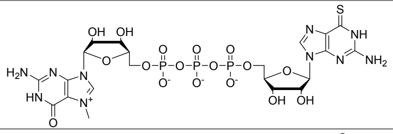	14.1 ± 1.7	[9]
**17.**	m^{7}G$_m$ppp^{6S}G	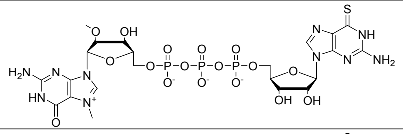	7.2 ± 1.1	[9]
**18.**	m^{7}G$_N$pppG	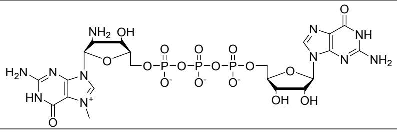	32.1 ± 5.9	[10]
**19.**	m^{7}GpCH$_2$ppG	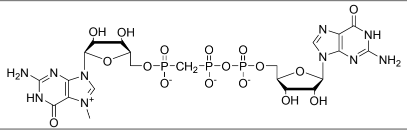	16.8 ± 3.3	[11]
**20.**	m^{7}Gpppm^{7}G	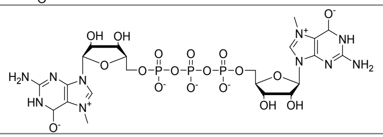	14.3 ± 2.1	[12]
**21.**	m^{7}Gp$_S$ppp$_S$m^{7}G<br>D3	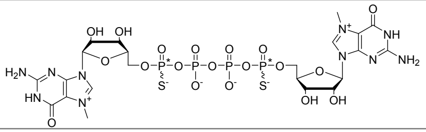	3.6 ± 0.4	[13]
**22.**	m^{7}G$_m$pppCH$_2$pG	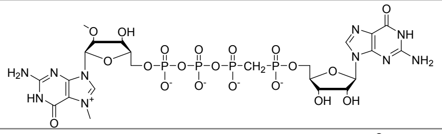	2.6 ± 0.4	[14]
**23.**	m^{7}GppCF$_2$ppG	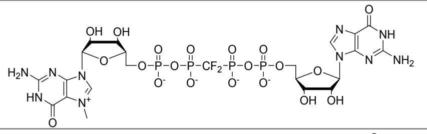	16.7 ± 3.1	[15]
**24.**	m^{7}GppCCl$_2$ppG	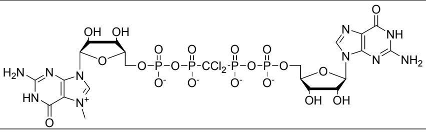	41.4 ± 6.6	[15]
**25.**	m^{7}GppCF$_2$ppm^{7}G	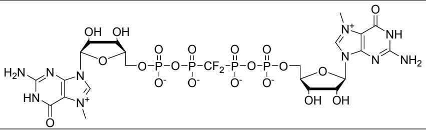	14.7 ± 2.6	unpublished

**26.**	$m^7GppCCl_2ppm^7G$	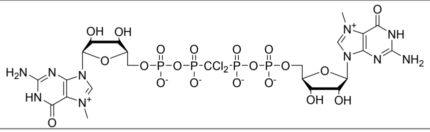	28.8 ± 4.1	unpublished
**27.**	$m^7GppCCppG$	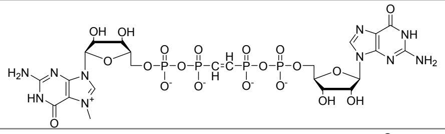	24.4 ± 4.0	unpublished
**28.**	$m^7GpppppG$	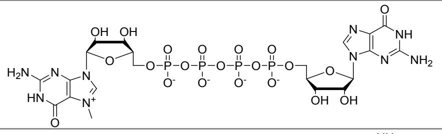	13.9 ± 2.5	[16]
**29.**	m^7GpppA	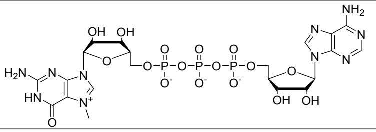	33.0 ± 5.9	[17] [18]
**30.**	GpppA	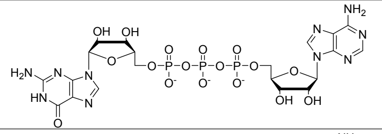	>>100	[18]
**31.**	m^7GpppA_m	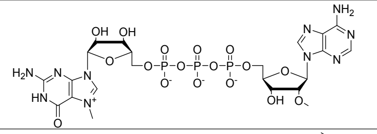	19.8 ± 3.6	unpublished
**32.**	$m^7Gppp(m^6A)$	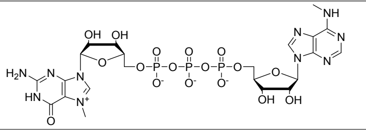	31.5 ± 5.9	unpublished
**33.**	Gpp-triazole-m^7G	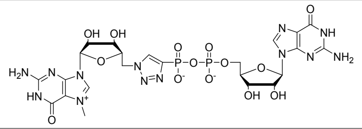	>>100	[19]
**34.**	Gppp-triazole-m^7G	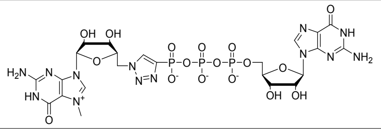	35.0 ± 5.3	[19]
**35.**	Gppp-SCH_2-triazole-m^7G	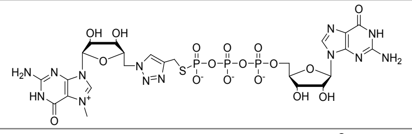	>>100	[19]
**36.**	m^7Gpp-triazole-G	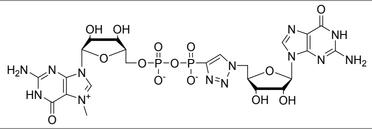	17.5 ± 2.6	[19]
**37.**	m^7Gpp-SCH_2-triazole-G	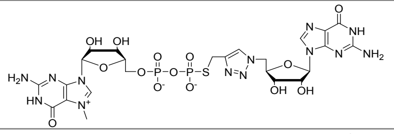	46.0 ± 6.8	[19]
**38.**	Gpp-SCH_2-triazole-m^7G	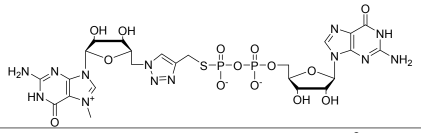	>>100	[19]
**39.**	m^7Gppp-SCH_2-triazole-G	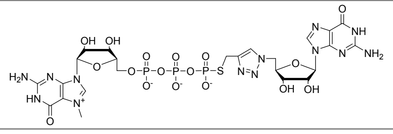	24.9 ± 4.8	[19]

**40.**	ApppA	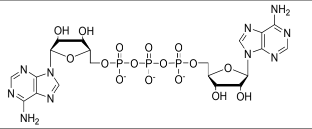	>>100	[7]
**41.**	Ap_Spp_SA	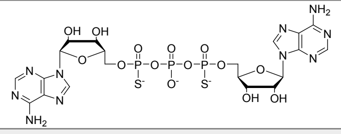	>>100	unpublished
	trinucleotides			
**42.**	m^7GpppApG	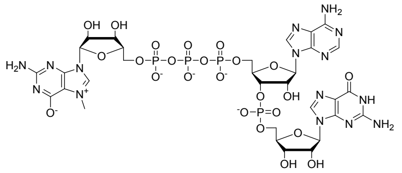	11.3 ± 2.0	[20]
**43.**	m^7Gpppm^6ApG	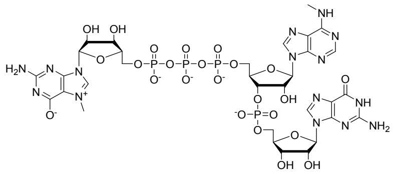	16.9 ± 3.2	[20]
**44.**	$m^7Gppp\ m^6A_mpG$	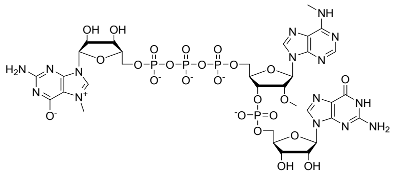	17.4 ± 3.0	[20]
**45.**	m^7GpppA_mpG	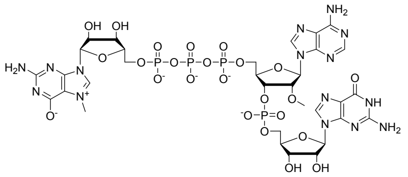	20.4 ± 3.4	[20]
**46.**	$m^7GppCH_2pA_mpG$	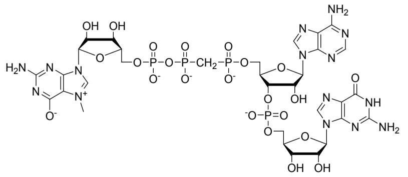	27.4 ± 6.2	[21]
**47.**	m^7GpppG_mpG	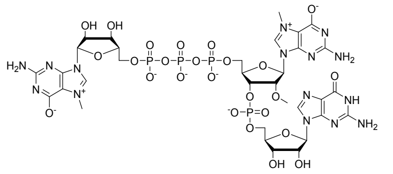	12.0 ± 2.4	[20]
**48.**	$m^7G_mpppG_mpG$	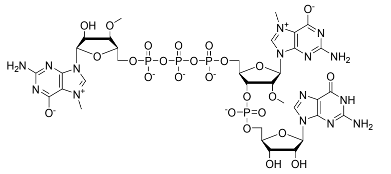	3.9 ± 0.5	unpublished

2. Supporting figures.

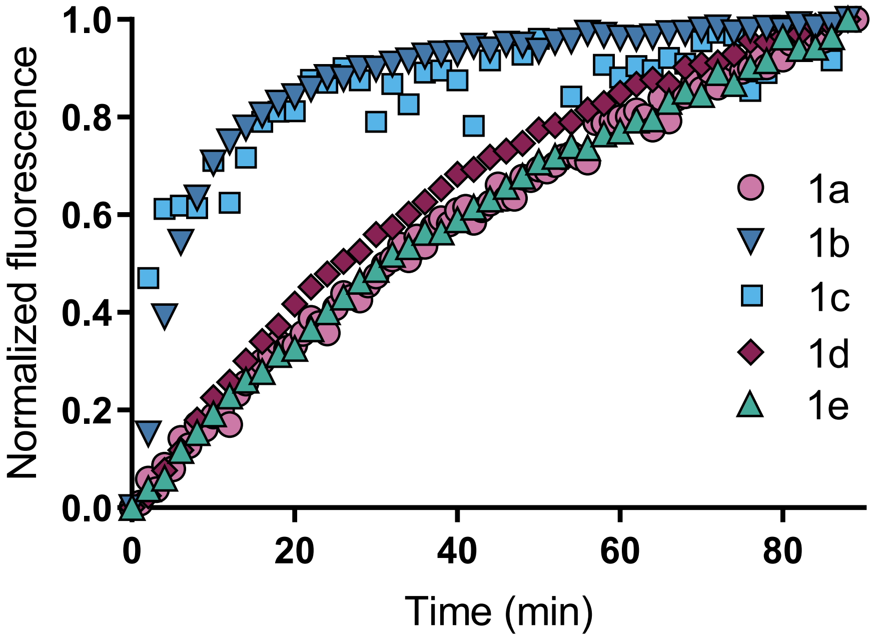

Figure S1. Relative kinetics of probe hydrolysis. To compare the relative susceptibility of probes to degradation by D9, the data shown in Figure 2C for each probe was transformed by normalizing the fluorescence intensities to the value at timepoint 90 min. The comparison showed that probes **1a** and **1b** were completely hydrolyzed within ~40 min, whereas the hydrolysis of all other probes was significantly slower and still not completed within 90 min. Reaction conditions: probe **1a–1e** (100 nM), D9 (50 nM) in 10 mM Tris·HCl containing 100 mM KOAc, 2 mM $MgCl_2$, and 0.5 mM $MnCl_2$, pH 7.5. For each probe florescence change at emission maximum was monitored (**1a** – exc.490 / em.512 nm; **1b, 1d, 1e** – 345 / 378 nm; **1c** – 420 / 489 nm).

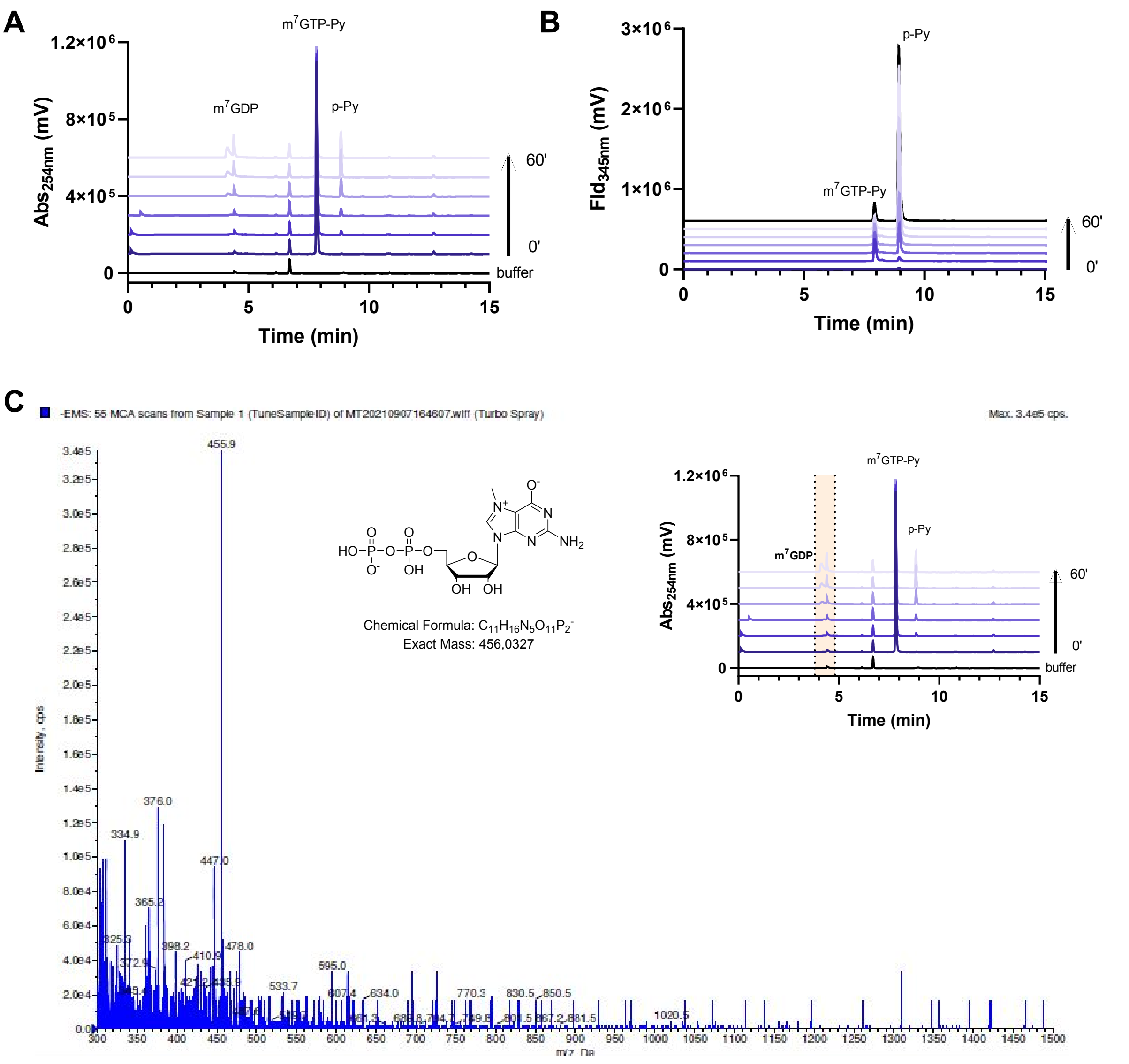
A
m7GTP-Py
m7GDP
p-Py
Abs254nm (mV)
1.2×10⁶
8×10⁵
4×10⁵
0
60'
0'
buffer
Time (min)
B
p-Py
m7GTP-Py
Fld345nm (mV)
3×10⁶
2×10⁶
1×10⁶
Time (min)
C
-EMS: 55 MCA scans from Sample 1 (TuneSampleID) of MT20210907164607.wiff (Turbo Spray)
Max. 3.4e5 cps.
455.9
376.0
334.9
447.0
365.2
478.0
398.2
595.0
533.7
770.3
830.5
850.5
1020.5
Intensity, cps
m/z, Da
HO-P-O-P-O
OH OH
NH2
Chemical Formula: C11H16N5O11P2-
Exact Mass: 456,0327
m7GTP-Py
m7GDP
p-Py
Abs254nm (mV)
Time (min)
buffer

D

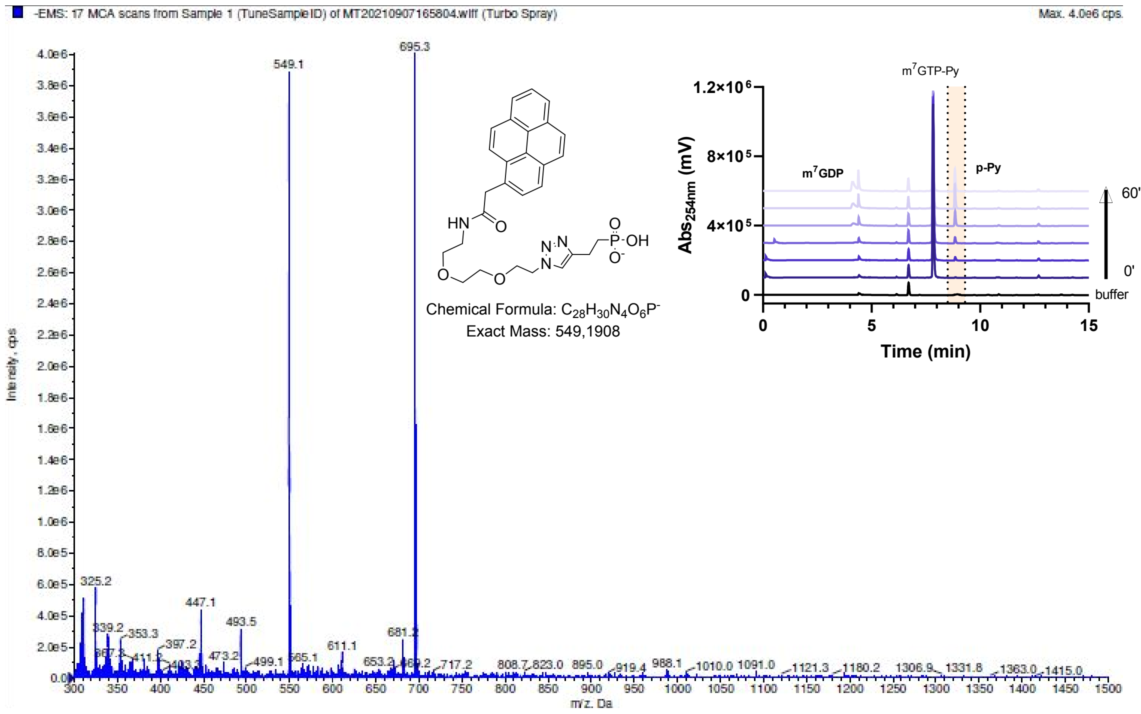

E

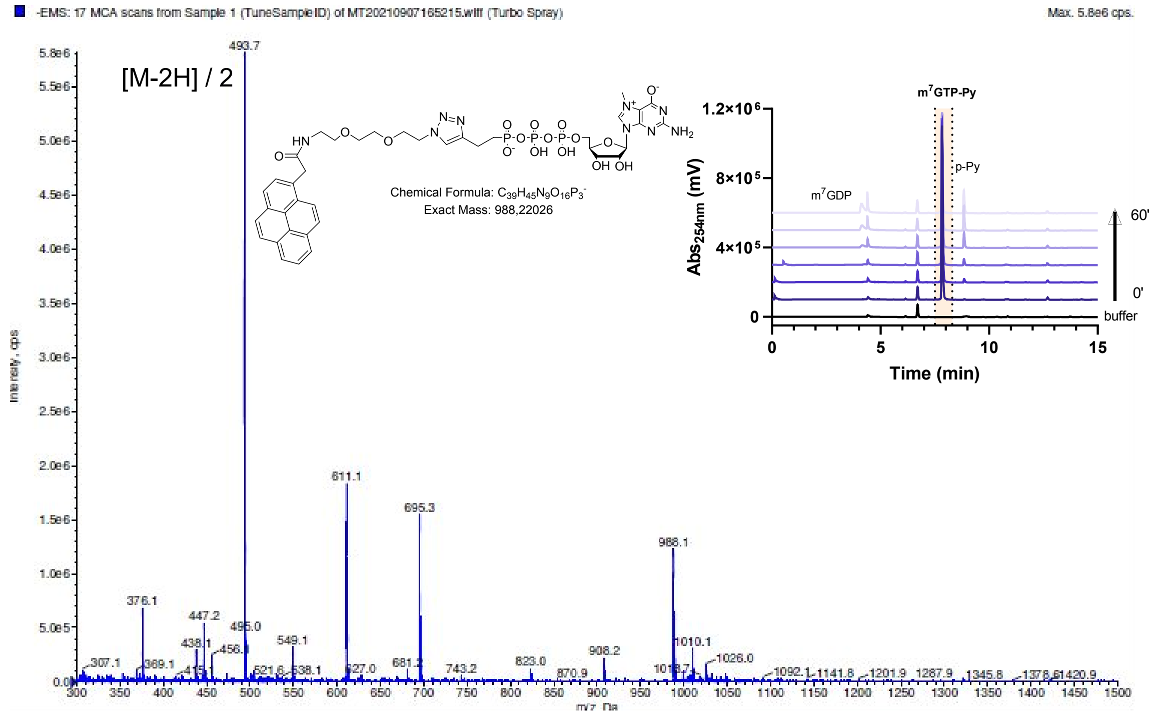

Figure S2. Hydrolysis of probe m^{7}GTP-Py (1b) monitored by RP HPLC (A, B) and mass spectrometry (C, D, E).

(A) Absorbance and (B) fluorescent RP HPLC profiles obtained during enzymatic reactions. Conditions: 100 µM **1b**, 25 nM D9 in 10 mM Tris·HCl pH 7.5 containing 100 mM KOAc, 2 mM DTT, 2 mM $MgCl_2$ and 0.3 mM $MnCl_2$; 30°C, 300 rpm. At different time points the reaction was thermally quenched, centrifuged and injected on HPLC. Hydrolysis products were collected during analysis and measured using mass spectrometry to identify the mass of the products (C, D, E).

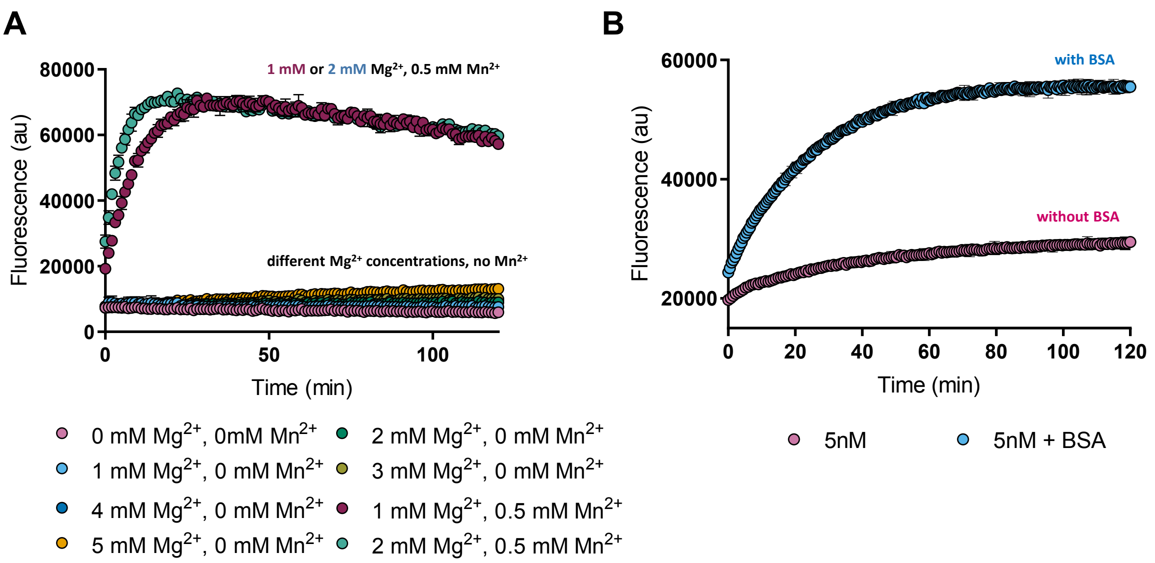

Figure S3. Selected results from HTS assay optimization.
(A) Conditions: probe **1b** (2 μM) was incubated for 2h with D9 enzyme (25 nM) in 10 mM Tris·HCl pH 7.5 containing 100 mM KOAc, 2 mM DTT and different magnesium and manganese concentrations were tested (see the legend); (B) Conditions: 2 μM **1b**, 25 nM D9 in 10 mM Tris·HCl pH 7.5 containing 100 mM KOAc, 2 mM DTT, 2 mM $MgCl_2$, 0.3 mM $MnCl_2$ and with (blue points) or without (pink points) the addition of 0.1% BSA.

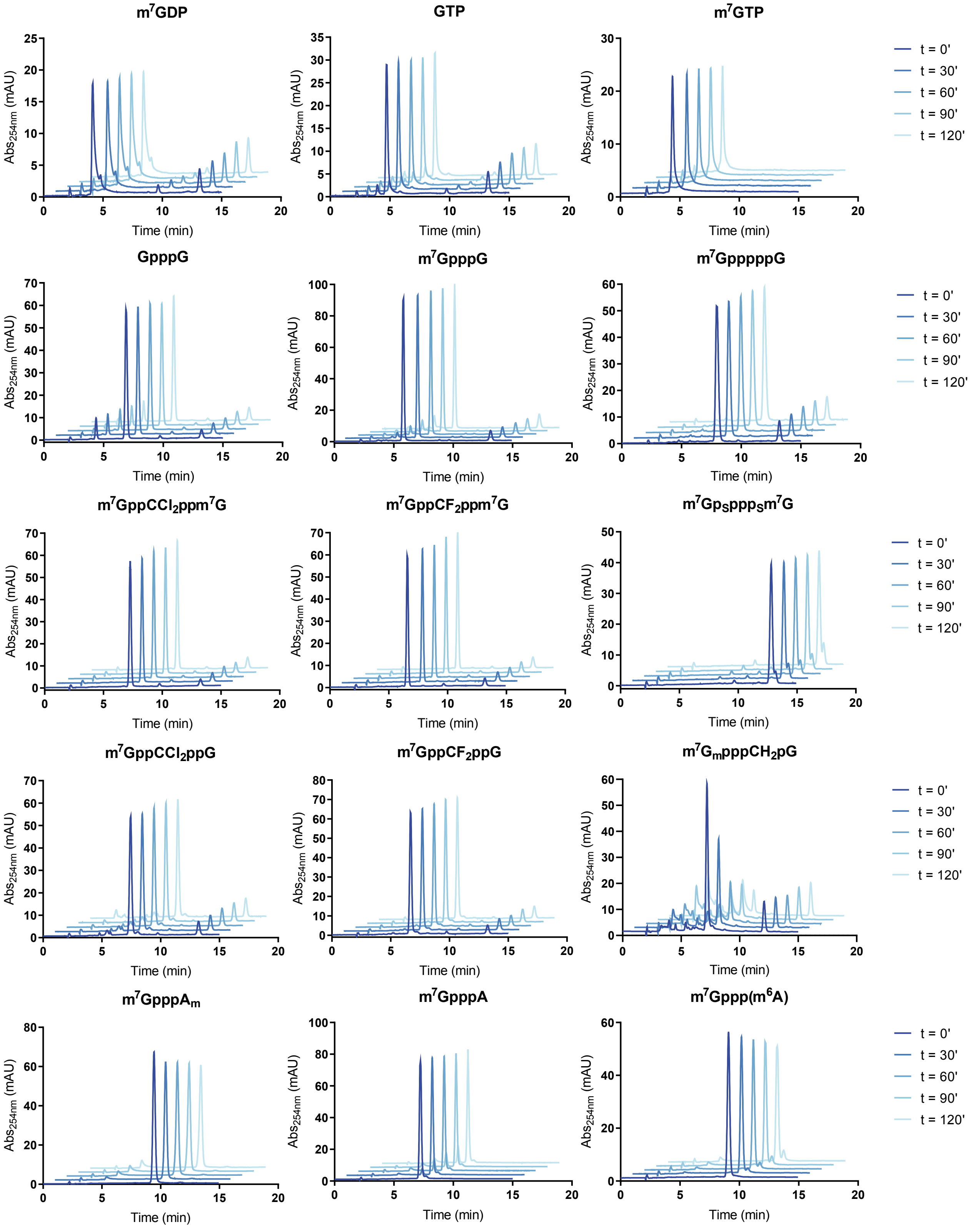

Figure S4. Analysis of cap analogs susceptibility to hydrolysis by D9 using RP HPLC. Conditions: 30 µM of cap analog, 25 nM D9 in 10 mM Tris·HCl pH 7.5 containing 100 mM KOAc, 2 mM DTT, 2 mM $MgCl_2$ and 0.3 mM $MnCl_2$, 30°C, 300 rpm. At different time points the reactions were thermally quenched, centrifuged and analyzed using RP HPLC.

A

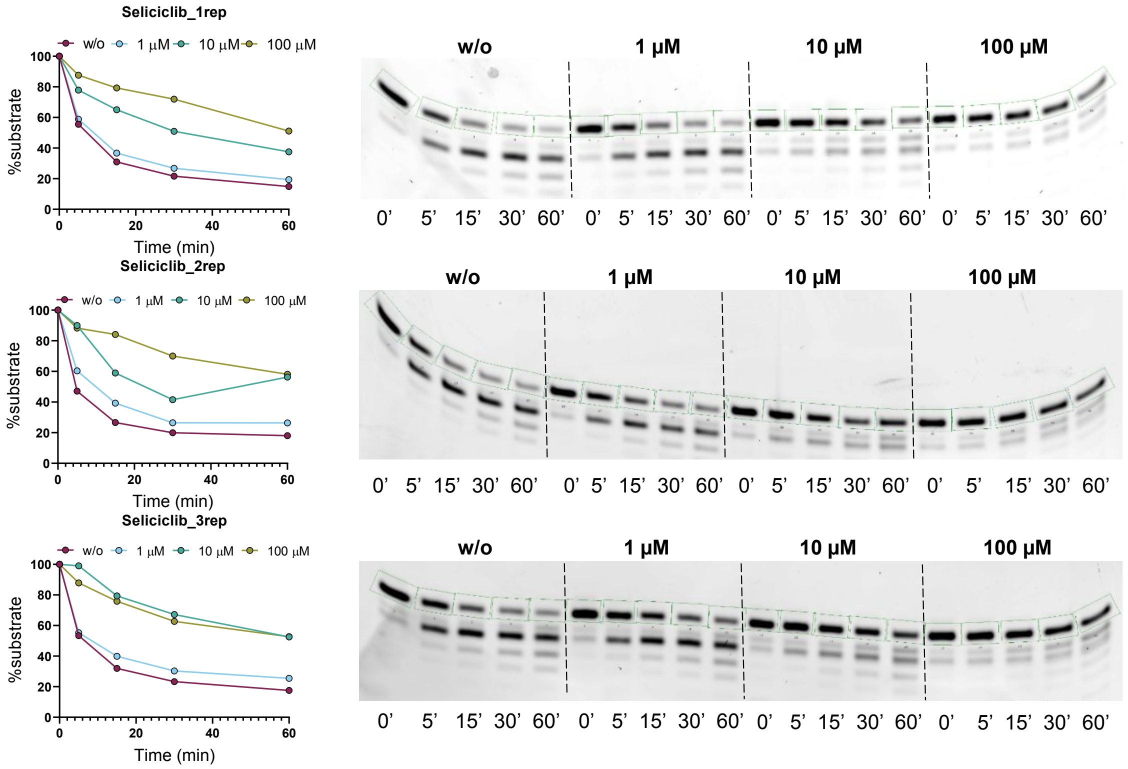
Seliciclib_1rep
Seliciclib_2rep
Seliciclib_3rep
w/o
1 μM
10 μM
100 μM
%substrate
Time (min)
0' 5' 15' 30' 60'

B

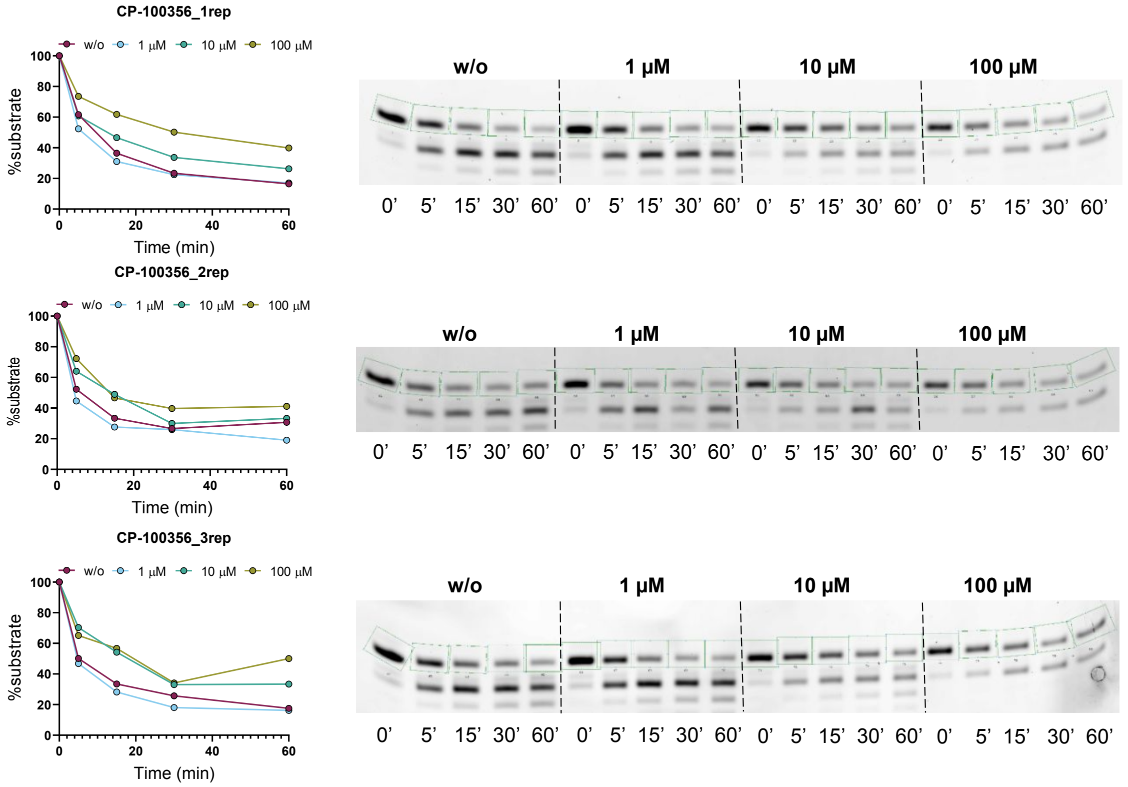
CP-100356_1rep
CP-100356_2rep
CP-100356_3rep
w/o
1 μM
10 μM
100 μM
%substrate
Time (min)
0' 5' 15' 30' 60'

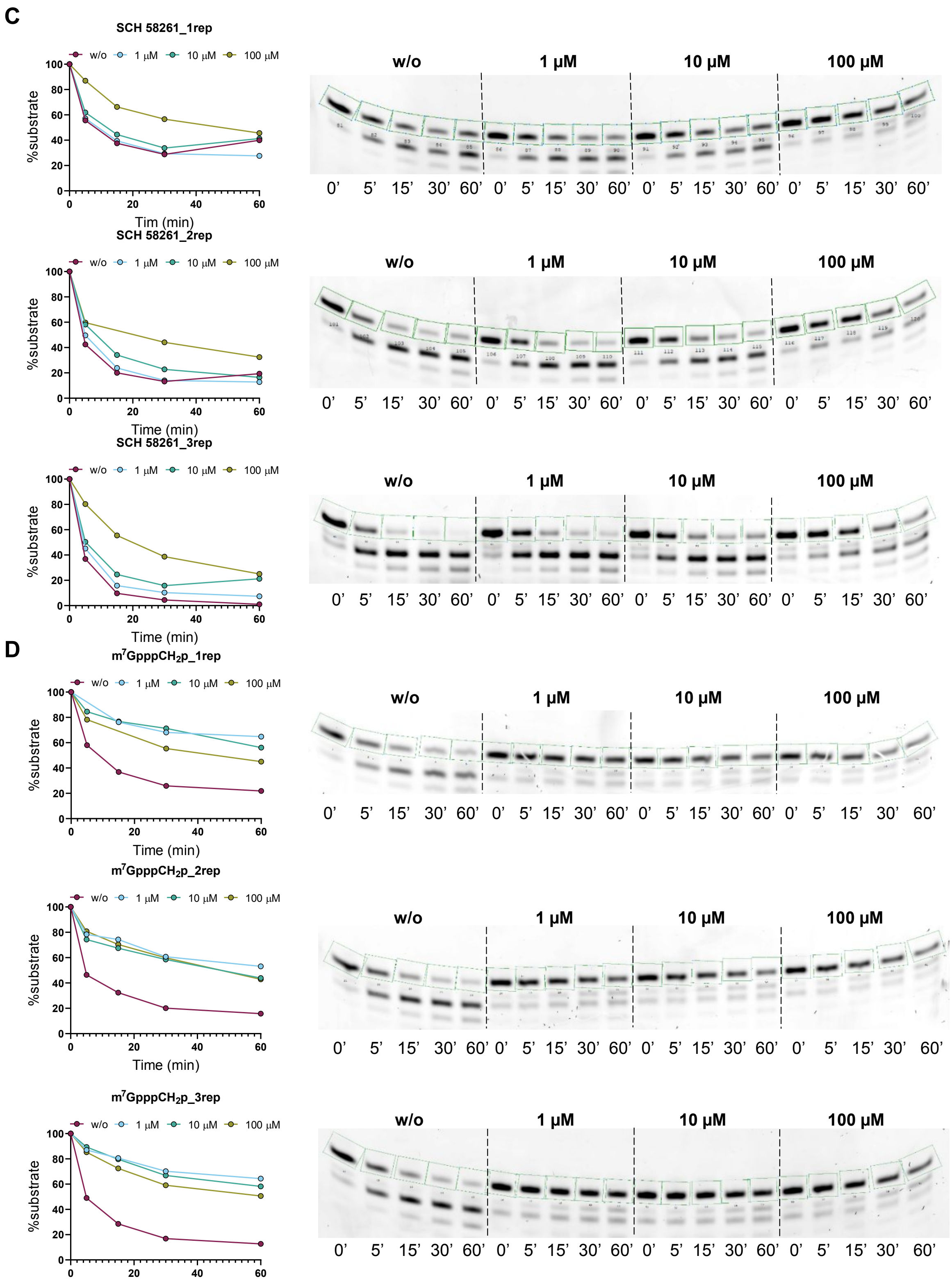

Figure S5. Verification of the identified hits in decapping assay on short RNA substrates. Reaction conditions: 20 ng of m^7Gppp-capped RNA (26 nt) was incubated for 1 h at 37°C with D9 enzyme (3 nM) without or with the presence of the tested inhibitor at 1, 10 or 100 µM in 10 mM MOPS·KOH pH 7.0 containing 100 mM KOAc, 2 mM DTT, 2 mM $MgCl_2$, 0.3 mM $MnCl_2$ and 0.1% BSA. Samples from different time points were analyzed by PAGE with SYBR Gold staining.

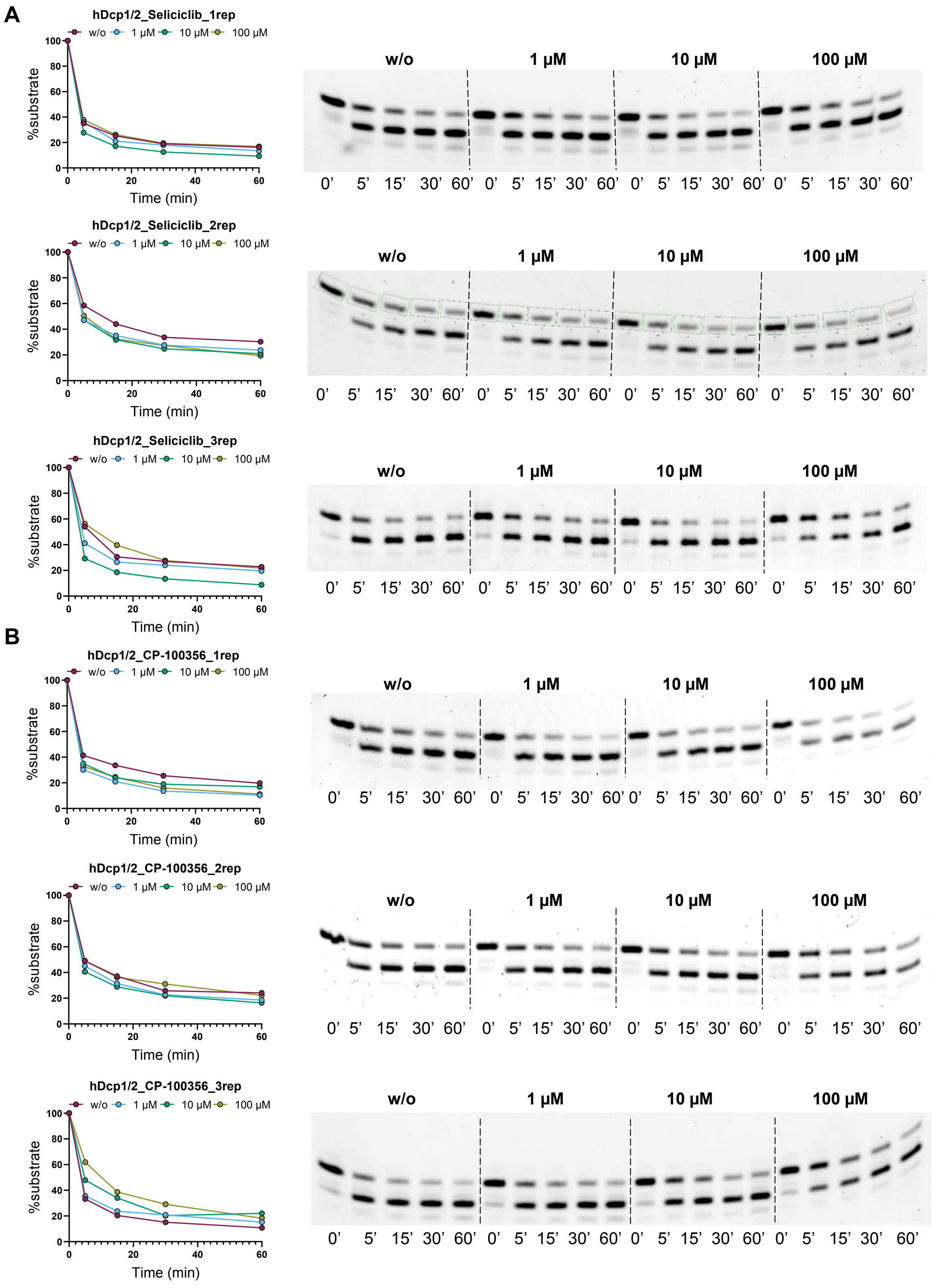
A
hDcp1/2_Seliciclib_1rep
hDcp1/2_Seliciclib_2rep
hDcp1/2_Seliciclib_3rep
B
hDcp1/2_CP-100356_1rep
hDcp1/2_CP-100356_2rep
hDcp1/2_CP-100356_3rep
w/o
1 µM
10 µM
100 µM
%substrate
Time (min)
0' 5' 15' 30' 60'

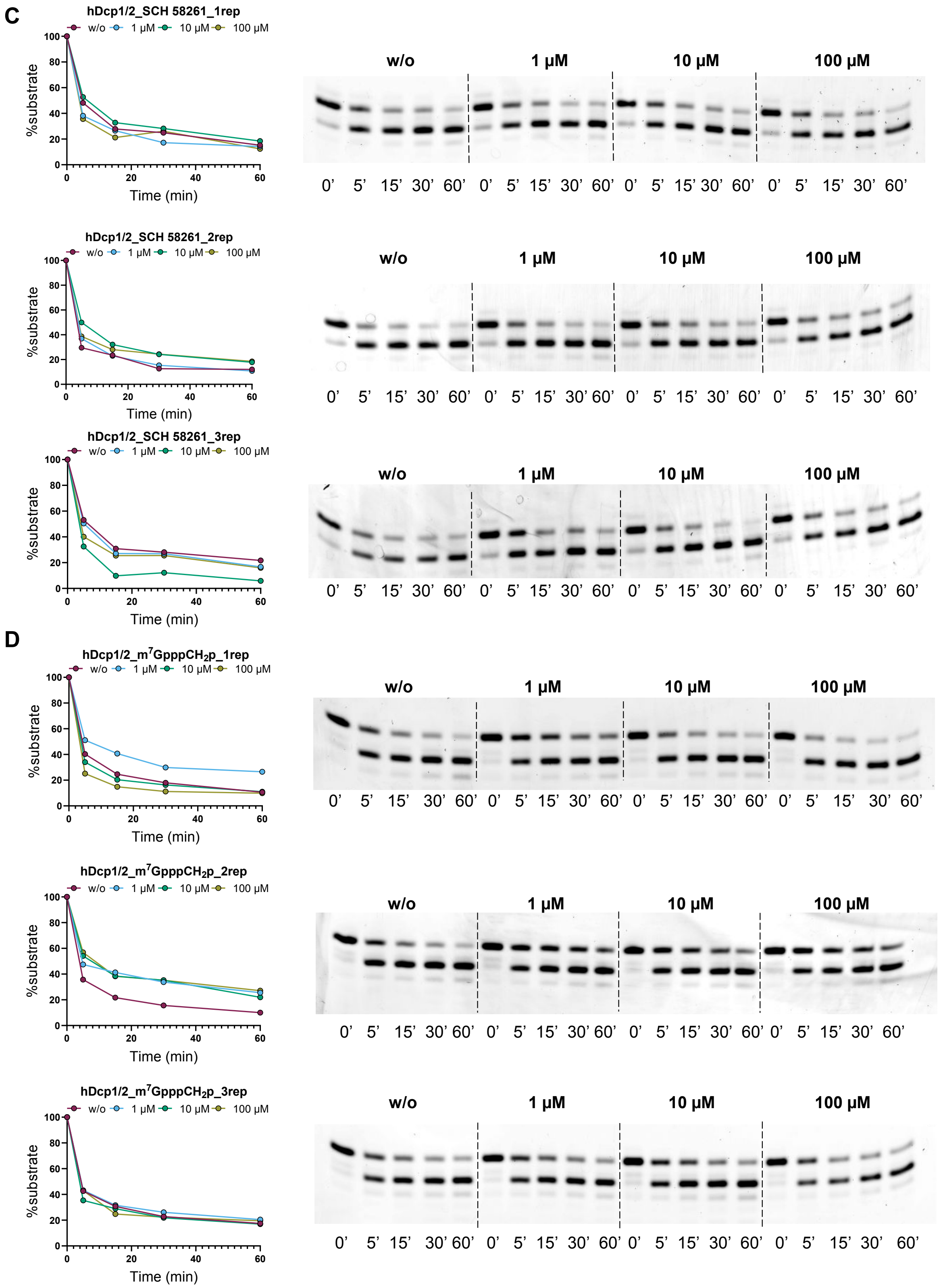

Figure S6. Verification of the selectivity of identified hits toward hDcp1/2 enzyme in decapping assay on short RNA substrates. Reaction conditions: 20 ng of m^7Gppp-capped RNA (26 nt) was incubated for 1 h at 37°C with hDcp1/2 complex (11 nM) without or with the presence of the tested inhibitor at 1, 10 or 100 µM in 50 mM Tris·HCl pH 8.0 containing 50 mM NH_4Cl, 0.01% Igepal, 1 mM DTT, 5 mM $MgCl_2$ and 0.1% BSA. Samples from different time points were analyzed by PAGE with SYBR Gold staining.

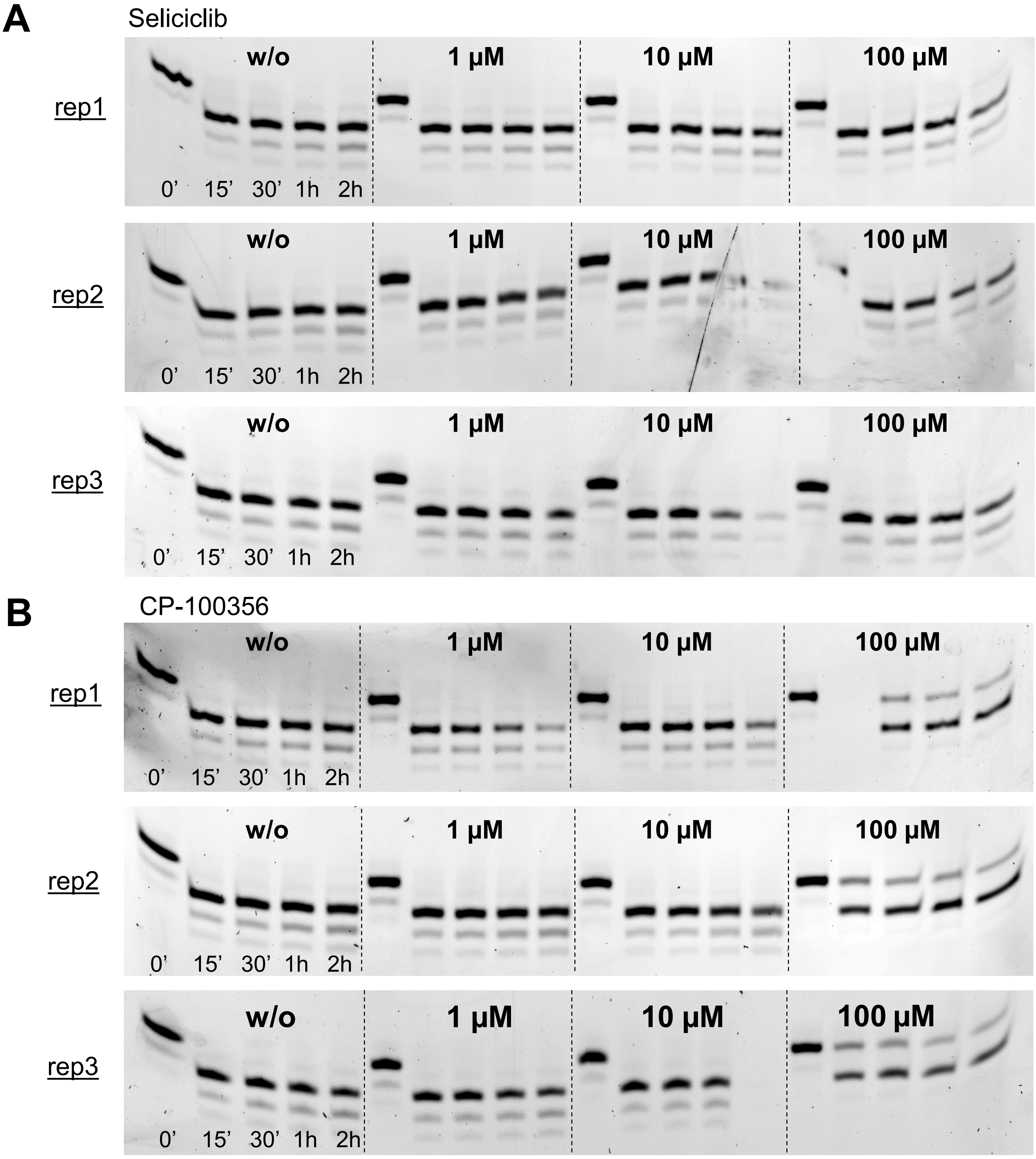
A
Seliciclib
w/o
1 µM
10 µM
100 µM
rep1
rep2
rep3
0'
15'
30'
1h
2h
B
CP-100356
w/o
1 µM
10 µM
100 µM
rep1
rep2
rep3
0'
15'
30'
1h
2h

C

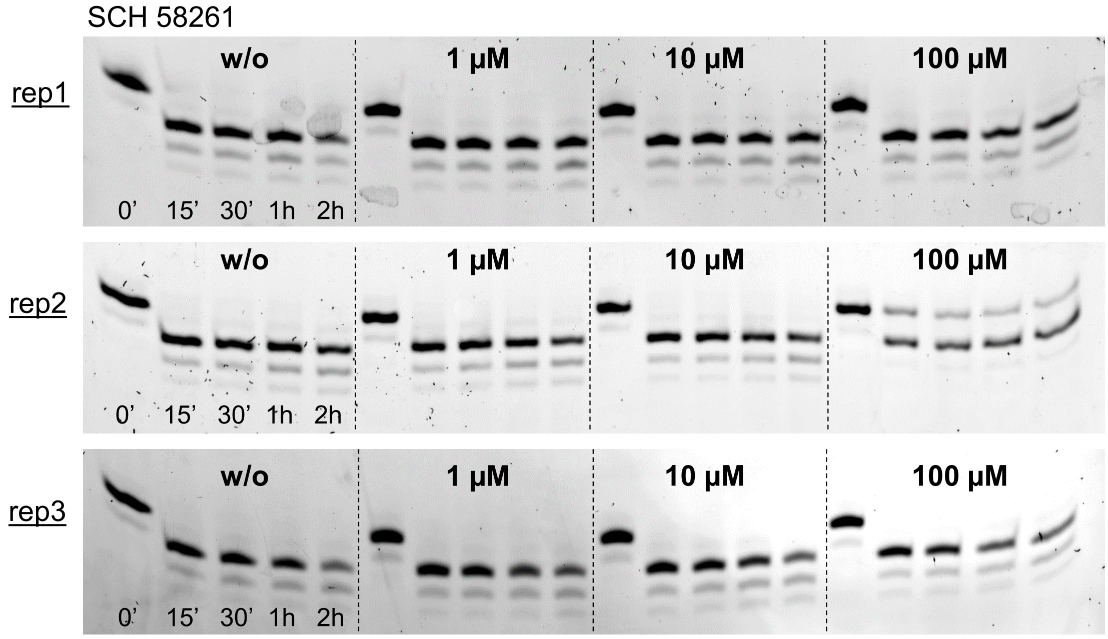

D

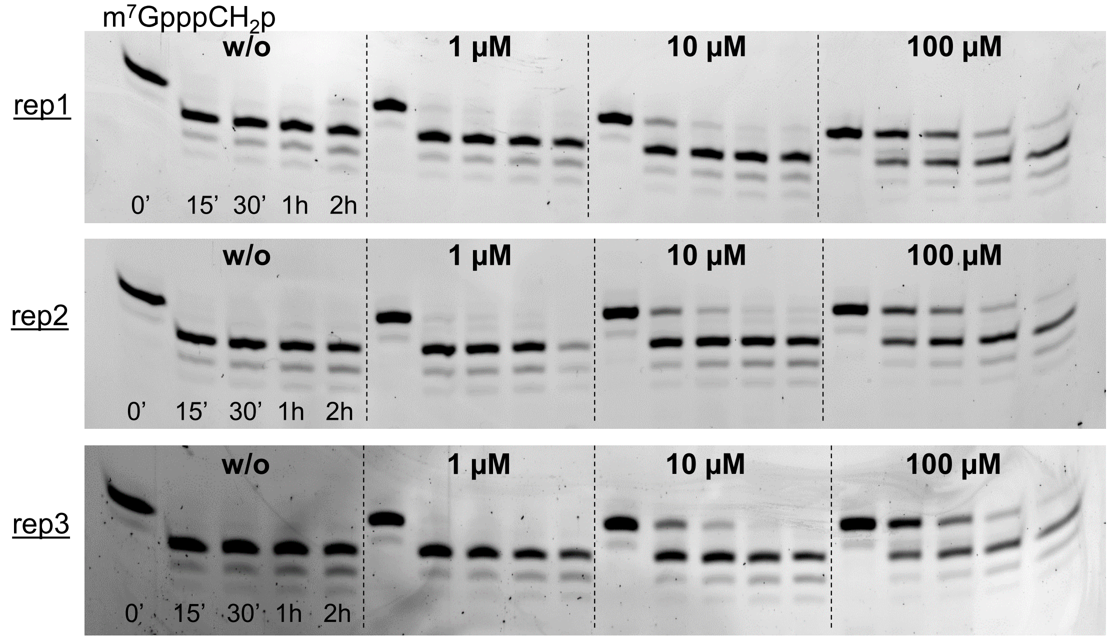

Figure S7. Verification of the selectivity of identified hits toward D10 enzyme in decapping assay on short RNA substrates. Reaction conditions: 20 ng of m7Gppp-capped RNA (26 nt) was incubated for 2h at 37°C with D10 enzyme (200 nM) without or with the presence of the tested inhibitor at 1, 10 or 100 µM in 10 mM MOPS·KOH pH 7.0 containing 100 mM KOAc, 2 mM DTT, 2 mM $MgCl_2$, 0.3 mM $MnCl_2$ and 0.1% BSA. Samples from different time points were analyzed by PAGE with SYBR Gold staining.

A)

ATGGGATCTTCGCACCATCATCACCATCACTCGTCCGGCCTGGTCCCGCGCGGTTCCCACATGT
CGGATAAGATTATTCATCTGACAGACGATAGCTTCGATACTGATGTTCTGAAGGCGGACGGAG
CGATCTTGGTAGACTTCTGGGCCGAATGGTGCGGCCCATGTAAGATGATTGCCCCCATTCTTGA
TGAAATTGCCGACGAATATCAAGGCAAGCTTACTGTCGCGAAGCTGAACATCGATCAAAACCC
CGGAACTGCCCCAAAATACGGTATTCGCGGCATCCCCACTTTATTATTATTCAAGAACGGAGAG
GTTGCAGCAACCAAAGTGGGAGCTTTATCTAAAGGTCAGCTTAAGGAATTTCTGGATGCCAACC
TGGCTGCTAGTGGAACGACAGAGAACTTGTACTTCCAGGGCAGCCAAAACTACGCTGGAGCGA
AATTTTCGGAGCCGCCTTCTCCTTCGGTTCTGCCGAAGCCGCCCAGTCACTGGGTCCCCGTGTC
GTTTAATCCTGGTGGAGGTGGTTCCGGAGGTGGAGGCAGCATGGAGACAAAGCGTGTGGAGA
TCCCTGGATCTGTCCTGGATGATCTTTGTTCGCGTTTCATCTTACATATTCCATCCGAAGAGCGC
GACAATGCAATTCGTGTTTGTTTCCAGATCGAGTTGGCCCACTGGTTCTACTTAGATTTTTACAT
GCAGAATACCCCTGGGTTACCCCAATGTGGAATCCGCGATTTCGCGAAAGCGGTATTTTCACA
CTGCCCGTTTTTGTTGCCTCAGGGGGAAGATGTCGAAAAAGTCTTGGATGAGTGGAAGGAGTA
TAAGATGGGGGTACCTACGTACGGAGCCATTATTTTGGACGAGACTCTTGAGAACGTATTACTT
GTCCAAGGCTATCTGGCGAAGTCCGGATGGGGATTCCCGAAAGGCAAGGTAAACAAGGAGGA
AGCGCCACATGACTGCGCGGCTCGCGAAGTTTTCGAAGAAACGGGTTTCGACATTAAAGATTA
CATCTGCAAAGACGACTATATCGAACTTCGTATTAACGACCAGCTTGCTCGCTTGTATATCATC
CCTGGGATTCCTAAGGATACTAAATTTAACCCAAAGACGCGCCGTGAGATTCGTAATATTGAAT
GGTTTAGTATCGAGAAATTGCCATGTCACCGCAACGATATGACCCCTAAAAGCAAACTGGGCTT
GGCTCCTAATAAATTTTTTATGGCTATCCCGTTTATTCGCCCATTGCGTGACTGGCTGTCACGCC
GCTAA

MGSSHHHHHHSSGLVPRGSHMSDKIIHLTDDSFDTDVLKADGAILVDFWAEWCGPCKMIAPILDEIA
DEYQGKLTVAKLNIDQNPGTAPKYGIRGIPTLLLFKNGEVAATKVGALSKGQLKEFLDANLAASGTT
ENLYFQGSQNYAGAKFSEPPSPSVLPKPPSHWVPVSFNPGGGGSGGGGSMETKRVEIPGSVLDDL
CSRFILHIPSEERDNAIRVCFQIELAHWFYLDFYMQNTPGLPQCGIRDFAKAVFSHCPFLLPQGEDVE
KVLDEWKEYKMGVPTYGAIILDETLENVLLVQGYLAKSGWGFPKGKVNKEEAPHDCAAREVFEETG
FDIKDYICKDDYIELRINDQLARLYIIPGIPKDTKFNPKTRREIRNIEWFSIEKLPCHRNDMTPKSKLGLA
PNKFFMAIPFIRPLRDWLSRR*

B)

ATGGCGCGTTCGATGGAATACAAGTTAATCTTAAACGGGAAAACTTTGAAAGGGGAAACGACT
ACGGAGGCCGTAGACGCAGCGACTGCGGAAAAAGTATTCAAACAATATGCTAATGATAATGGC
GTTGATGGGGAATGGACTTATGACGATGCCACAAAGACATTCACCGTTACGGAAATCCCTACG
ACGGAGAACTTATATTTTCAGGGTTCGATGGAGGCTTTGTCCCGTGCAGGACAGGAAATGAGTT
TGGCTGCCCTGAAGCAACATGACCCATACATCACGAGTATTGCAGATTTGACGGGACAGGTCG
CGTTATACACATTTTGTCCAAAGGCAAACCAATGGGAGAAGACAGATATTGAGGGTACATTGTT
CGTTTATCGTCGCTCTGCTTCGCCGTACCACGGGTTTACGATTGTTAACCGTTTGAATATGCACA
ATCTGGTAGAGCCAGTCAACAAAGACCTGGAATTTCAACTTCACGAACCGTTTTTGTTGTACCG
CAACGCATCTCTGTCAATCTATTCCATCTGGTTCTACGACAAAAATGATTGCCATCGCATCGCG
AAATTGATGGCTGACGTAGTCGAGGAAGAAACCCGCCGCTCCCAGCAGTAA

MARSMEYKLILNGKTLKGETTTEAVDAATAEKVFKQYANDNGVDGEWTYDDATKTFTVTEIPTTENL
YFQGSMEALSRAGQEMSLAALKQHDPYITSIADLTGQVALYTFCPKANQWEKTDIEGTLFVYRRSA
SPYHGFTIVNRLNMHNLVEPVNKDLEFQLHEPFLLYRNASLSIYSIWFYDKNDCHRIAKLMADVVEEE
TRFRSQQ*

● 6 x His ● TEV site ●Dcp2 protein

●Thrombin site ● PNRC2 protein ● Gb1 protein

● Trx protein ●Linker (G4S)2 ●Dcp1 protein

Figure S8. Nucleic and amino acid sequence of human Dcp1-Dcp2 complex used in this study. (A) Nucleic and amino acid sequence of 6xHis-Trx-PNRC2-Dcp2; (B) Nucleic and amino acid sequence of Gb1- Dcp1.

3. Raw data: HPLC profiles and HRMS spectra for newly synthesized compounds.

m^7GTPγS-BODIPY-FL (1a)

Formula

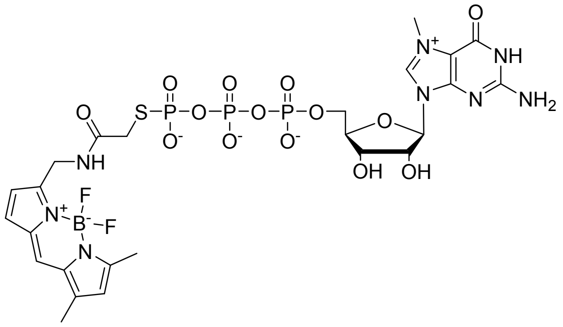

HPLC profile

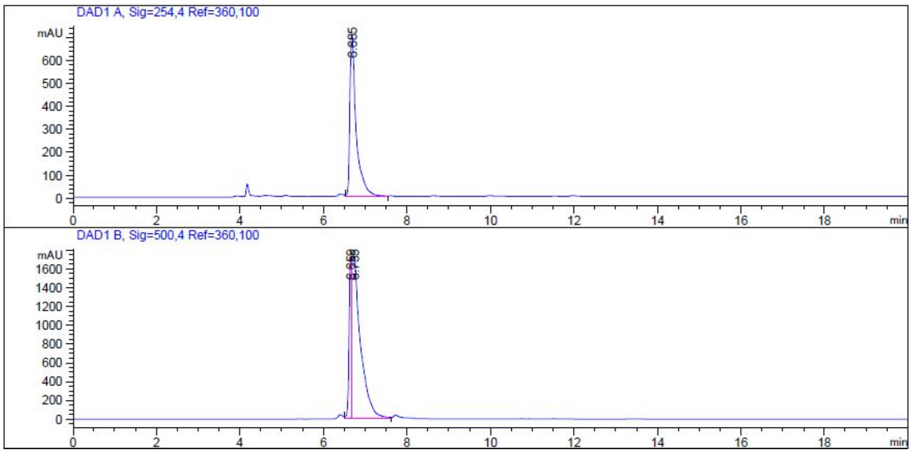

HRMS (-)ESI

HRMS (ESI-) Teor. m/z dla $C_{25}H_{31}BF_{2}N_{8}O_{14}P_{3}S^{-}$ $[M\text{-}H]^{-}$ 841.09599, recorded 841.09761

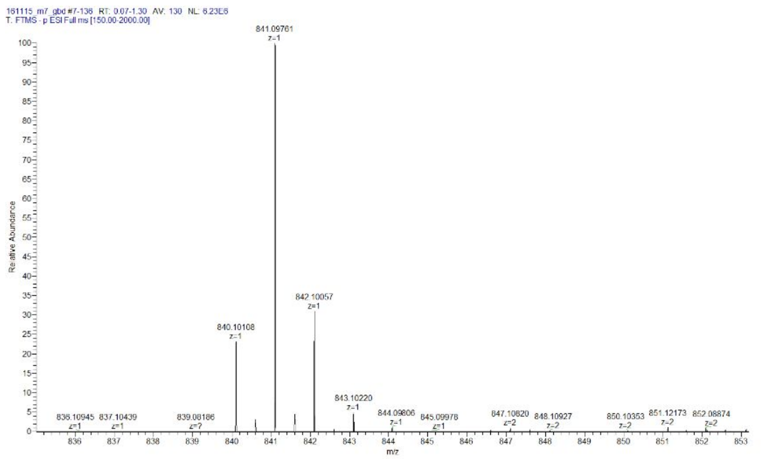

m⁷GTP-Pe (1c)

Formula

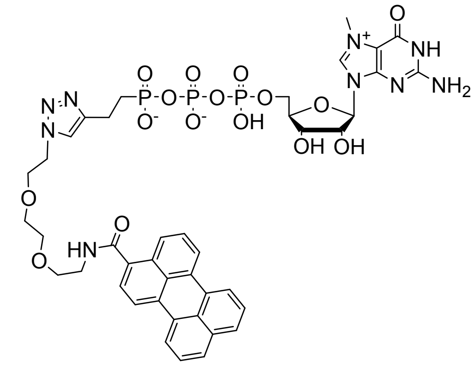

HPLC profile

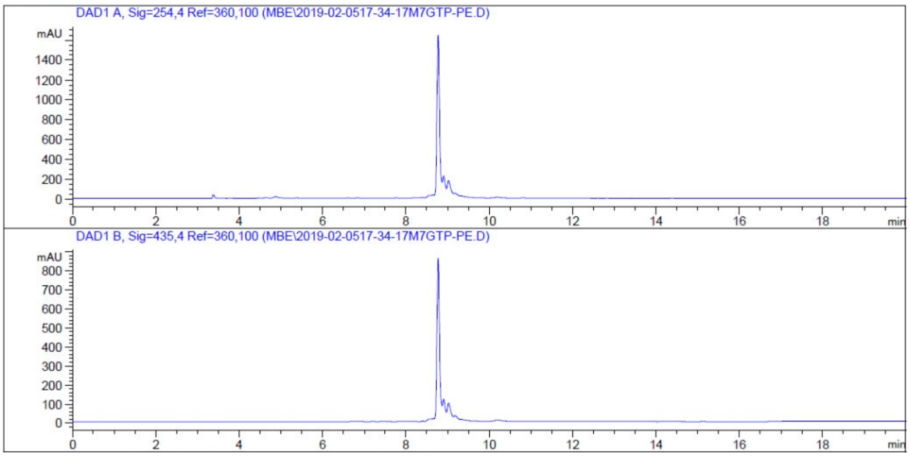

HRMS (-)ESI

HRMS (ESI-) Teor. m/z dla $C_{42}H_{45}N_9O_{16}P_3^-$ $[M-H]^-$ 1024.22026, recorded 1024.22208

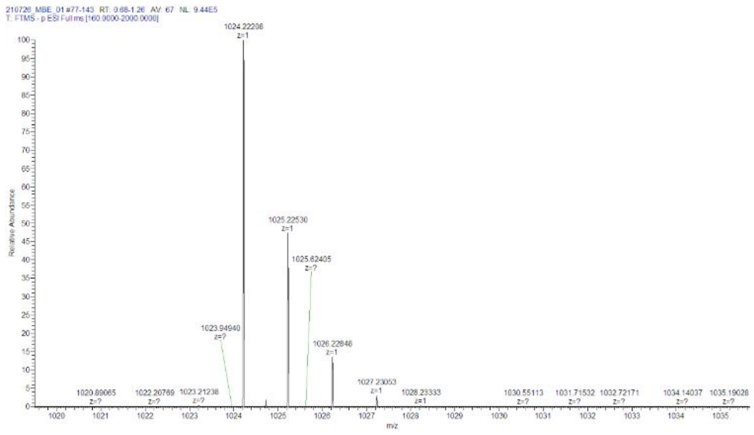

Supporting References

1. Darzynkiewicz, E.; Ekiel, I.; Tahara, S.M.; Seliger, L.S.; Shatkin, A.J. Chemical synthesis and characterization of 7-methylguanosine cap analogs. *Biochemistry* **1985**, *24*, 1701-1707, doi:10.1021/bi00328a020.
2. Strenkowska, M.; Wanat, P.; Ziemniak, M.; Jemielity, J.; Kowalska, J. Preparation of Synthetically Challenging Nucleotides Using Cyanoethyl P-Imidazolides and Microwaves. *Organic Letters* **2012**, *14*, 4782-4785, doi:10.1021/ol302071f.
3. Kowalska, J.; Lukaszewicz, M.; Zuberek, J.; Ziemniak, M.; Darzynkiewicz, E.; Jemielity, J. Phosphorothioate analogs of m(7)GTP are enzymatically stable inhibitors of cap-dependent translation. *Bioorganic & Medicinal Chemistry Letters* **2009**, *19*, 1921-1925, doi:10.1016/j.bmcl.2009.02.053.
4. Szczepaniak, S.A.; Zuberek, J.; Darzynkiewicz, E.; Kufel, J.; Jemielity, J. Affinity resins containing enzymatically resistant mRNA cap analogs-a new tool for the analysis of cap-binding proteins. *Rna* **2012**, *18*, 1421-1432, doi:10.1261/rna.032078.111.
5. Jemielity, J.; Pietrowska-Borek, M.; Starzynska, E.; Kowalska, J.; Stolarski, R.; Guranowski, A.; Darzynkiewicz, E. Synthesis and enzymatic characterization of methylene analogs of adenosine 5 '-tetraphosphate (P(4)A). *Nucleosides Nucleotides & Nucleic Acids* **2005**, *24*, 589-593, doi:10.1081/ncn-200061911.
6. Darzynkiewicz, E.; Stepinski, J.; Tahara, S.M.; Stolarski, R.; Ekiel, I.; Haber, D.; Neuvonen, K.; Lehikoinen, P.; Labadi, I.; Lonnberg, H. Synthesis, conformation and hydrolytic stability of P1,P3-dinucleoside triphosphates related to messenger-RNA 5'-cap, and comparative kinetic-studies on their nucleoside and nucleoside monophosphate analogs. *Nucleosides & Nucleotides* **1990**, *9*, 599-618, doi:10.1080/07328319008045191.
7. Pietrowska-Borek, M.; Wojdyla-Mamon, A.; Dobrogojski, J.; Mlynarska-Cieslak, A.; Baranowski, M.R.; Dabrowski, J.M.; Kowalska, J.; Jemielity, J.; Borek, S.; Pedreno, M.A.; et al. Purine and pyrimidine dinucleoside polyphosphates differentially affect the phenylpropanoid pathway in Vitis vinifera L. cv. Monastrell suspension cultured cells. *Plant Physiology and Biochemistry* **2020**, *147*, 125-132, doi:10.1016/j.plaphy.2019.12.015.
8. Stepinski, J.; Waddell, C.; Stolarski, R.; Darzynkiewicz, E.; Rhoads, R.E. Synthesis and properties of mRNAs containing the novel "anti-reverse" cap analogs 7-methyl(3 '-O-methyl)GpppG and 7-methyl(3 '-deoxy)GpppG. *Rna* **2001**, *7*, 1486-1495.
9. Nowakowska, M.; Kowalska, J.; Martin, F.; d'Orchymont, A.; Zuberek, J.; Lukaszewicz, M.; Darzynkiewicz, E.; Jemielity, J. Cap analogs containing 6-thioguanosine - reagents for the synthesis of mRNAs selectively photo-crosslinkable with cap-binding biomolecules. *Organic & Biomolecular Chemistry* **2014**, *12*, 4841-4847, doi:10.1039/c4ob00059e.
10. Jemielity, J.; Lukaszewicz, M.; Kowalska, J.; Czarnecki, J.; Zuberek, J.; Darzynkiewicz, E. Synthesis of biotin labelled cap analogue - incorporable into mRNA transcripts and promoting cap-dependent translation. *Organic & Biomolecular Chemistry* **2012**, *10*, 8570-8574, doi:10.1039/c2ob26060c.
11. Kalek, M.; Jemielity, J.; Grudzien, E.; Zuberek, J.; Bojarska, E.; Cohen, L.S.; Stepinski, J.; Stolarski, R.; Davis, R.E.; Rhoads, R.E.; et al. Synthesis and biochemical properties of novel mRNA 5 ' cap analogs resistant to enzymatic hydrolysis. *Nucleosides Nucleotides & Nucleic Acids* **2005**, *24*, 615-621, doi:10.1081/ncn-200060091.
12. Stepinski, J.; Bretner, M.; Jankowska, M.; Felczak, K.; Stolarski, R.; Wieczorek, Z.; Cai, A.L.; Rhoads, R.E.; Temeriusz, A.; Haber, D.; et al. Synthesis and properties of P(1),P(2), P(1),P(3) and P(1),P(4), dinucleoside diphosphate, triphosphate and tetraphosphate messenger-RNA 5'-cap analogs. *Nucleosides & Nucleotides* **1995**, *14*, 717-721.
13. Ziemniak, M.; Kowalska, J.; Lukaszewicz, M.; Zuberek, J.; Wnek, K.; Darzynkiewicz, E.; Jemielity, J. Phosphate-modified analogues of m(7)GTP and m(7)Gppppm(7)G-

Synthesis and biochemical properties. *Bioorganic & Medicinal Chemistry* **2015**, *23*, 5369-5381, doi:10.1016/j.bmc.2015.07.052.
14. Rydzik, A.M.; Lukaszewicz, M.; Zuberek, J.; Kowalska, J.; Darzynkiewicz, Z.M.; Darzynkiewicz, E.; Jemielity, J. Synthetic dinucleotide mRNA cap analogs with tetraphosphate 5 ',5 ' bridge containing methylenebis(phosphonate) modification. *Organic & Biomolecular Chemistry* **2009**, *7*, 4763-4776, doi:10.1039/b911347a.
15. Rydzik, A.M.; Warminski, M.; Sikorski, P.J.; Baranowski, M.R.; Walczak, S.; Kowalska, J.; Zuberek, J.; Lukaszewicz, M.; Nowak, E.; Claridge, T.D.W.; et al. mRNA cap analogues substituted in the tetraphosphate chain with CX2: identification of O-to-CCl2 as the first bridging modification that confers resistance to decapping without impairing translation. *Nucleic Acids Research* **2017**, *45*, 8661-8675, doi:10.1093/nar/gkx569.
16. Jemielity, J.; Fowler, T.; Zuberek, J.; Stepinski, J.; Lewdorowicz, M.; Niedzwiecka, A.; Stolarski, R.; Darzynkiewicz, E.; Rhoads, R.E. Novel "anti-reverse" cap analogs with superior translational properties. *Rna* **2003**, *9*, 1108-1122, doi:10.1261/rna.5430403.
17. Sawai, H.; Wakai, H.; Shimazu, M. Facile synthesis of cap portion of messenger-RNA by Mn(II) ion-catalyzed pyrophosphate formation in aqueous-solution. *Tetrahedron Letters* **1991**, *32*, 6905-6906, doi:10.1016/0040-4039(91)80440-h.
18. Guranowski, A.; Starzynska, E.; Bojarska, E.; Stepinski, J.; Darzynkiewicz, E. Dinucleoside 5',5'''-P-1,P-3-triphosphate hydrolase from yellow lupin (Lupinus luteus) seeds: Purification to homogeneity and hydrolysis of mRNA 5'-cap analogs. *Protein Expression and Purification* **1996**, *8*, 416-422, doi:10.1006/prep.1996.0119.
19. Walczak, S.; Nowicka, A.; Kubacka, D.; Fac, K.; Wanat, P.; Mroczek, S.; Kowalska, J.; Jemielity, J. A novel route for preparing 5 ' cap mimics and capped RNAs: phosphate-modified cap analogues obtained via click chemistry. *Chemical Science* **2017**, *8*, 260-267, doi:10.1039/c6sc02437h.
20. Sikorski, P.J.; Warminski, M.; Kubacka, D.; Ratajczak, T.; Nowis, D.; Kowalska, J.; Jemielity, J. The identity and methylation status of the first transcribed nucleotide in eukaryotic mRNA 5 ' cap modulates protein expression in living cells. *Nucleic Acids Research* **2020**, *48*, 1607-1626, doi:10.1093/nar/gkaa032.
21. Peters, J.K.; Tibble, R.W.; Warminski, M.; Jemielity, J.; Gross, J.D. Structure of the poxvirus decapping enzyme D9 reveals its mechanism of cap recognition and catalysis. *Structure* **2022**, *30*, 1-12, doi:10.1016/j.str.2022.02.012.
